# Evidence for Ruthenium(II) Peralkene Complexes as Catalytic Species during the Isomerization of Terminal Alkenes in Solution

**DOI:** 10.1021/acs.inorgchem.3c00967

**Published:** 2023-07-03

**Authors:** Sergio Sanz-Navarro, Jordi Ballesteros-Soberanas, Aarón Martínez-Castelló, Antonio Doménech-Carbó, Juan Carlos Hernández-Garrido, Jose Pedro Cerón-Carrasco, Marta Mon, Antonio Leyva-Pérez

**Affiliations:** † Instituto de Tecnología Química, Universitat Politècnica de València−Consejo Superior de Investigaciones Científicas, Avda. de los Naranjos s/n, 46022 Valencia, Spain; ‡ Zschimmer & Schwarz Spain, CTRA. CV-20, KM. 3.200. APDO. 118, 12540 Villareal, Spain; § Departament de Química Analítica, Universitat de Valencia, Dr Moliner, 50, Burjassot, 46100 Valencia, Spain; ∥ Departamento de Ciencia de los Materiales e Ingeniería Metalúrgica y Química Inorgánica, Facultad de Ciencias, Universidad de Cádiz, Campus Universitario Puerto Real, Puerto Real 11510, Cádiz, Spain; ⊥ Centro Universitario de la Defensa, Universidad Politécnica de Cartagena, Base Aérea de San Javier, C/Coronel López Peña S/N, Santiago de La Ribera, 30720 Murcia, Spain

## Abstract

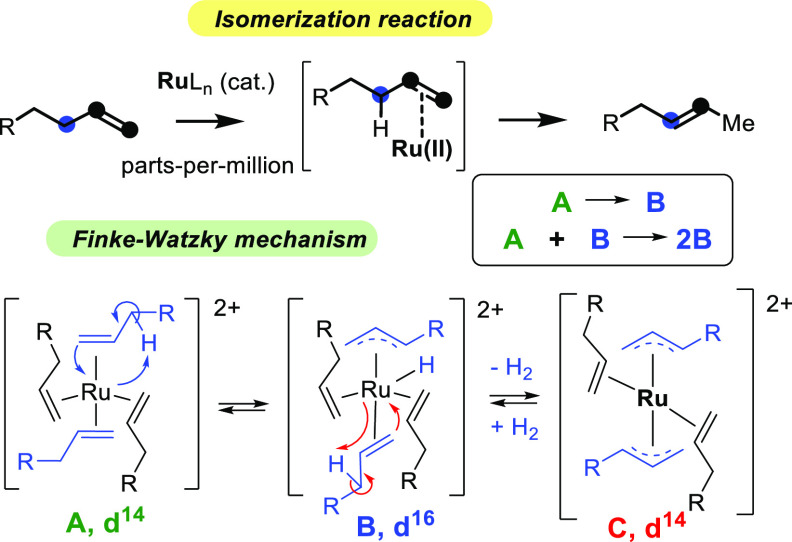

The isomerization (chain-walking) reaction of terminal to internal alkenes is catalyzed by part-per-million amounts of practically any Ru source when the reaction is carried out with a neat terminal alkene. Here, we provide evidence that the soluble starting Ru sources evolve to catalytically active *per*alkene Ru(II) species under reaction conditions. These species may also explain the isomerization products found during other Ru-catalyzed alkene processes, i.e., alkene metathesis reactions. A Finke–Watzky mechanism for catalyst formation is consistent with the evidence obtained.

## Abstract 2

The isomerization reaction of terminal to internal alkenes is plausibly catalyzed by part-per-million amounts of per-alkene Ru(II) complexes formed in situ from a variety of Ru sources. The reaction proceeds without any additive or solvent, and a Finke−Watzky mechanism for the catalyst formation is in operation here. The in situ transformation of Grubbs-type catalyst explains the commonly observed isomerization of alkenes during metathesis reactions.

## Introduction

The isomerization of terminal alkenes is the method of choice in industry to prepare internal alkenes.^1,2^ Terminal alkenes are cheap raw materials, particularly linear alkenes coming from controlled ethylene oligomerization reactions. However, the current industrial isomerization process for small alkenes employs zeolite-type solid catalysts at high temperatures, which produce branching and other acid-triggered, undesired byreactions. Rh salts are used in a homogeneous phase for bigger and more complex alkenes; however, Rh metal is extremely expensive and difficult to recover in solution.^3^ Thus, cheap catalysts able to perform the isomerization of alkenes without acid-catalyzed byreactions are of high interest.^4^ Among the different alternatives recently reported, we have shown that part-per-million (ppm) amounts of any Ru species available, except high-valent Ru(IV) and Ru(VI) species, catalyze the isomerization of a myriad of terminal alkenes into the corresponding internal alkenes, provided that the reaction is run solventless and at reaction temperature >150 °C.^5^ Here, we provide experimental and theoretical pieces of evidence that, regardless of the starting Ru species employed, the terminal alkene reactant displaces the ligands/anions of the Ru(II) complex/salt to generate *per*alkene Ru(II) species which catalyzes the isomerization reaction with great efficiency [turnover frequency (TOF) up to 10^8^ h^–1^]. The results also strongly support that the Ru(II) catalyst follows a Finke–Watzky catalyst formation mechanism^6,7^ and triggers a non-dissociative pathway (chain-walking) for alkene isomerization and that the *per*alkene Ru(II) active species excludes the internal alkene product of reacting further.

## Experimental Methods^8^

### Materials

All chemicals and Ru salts and complexes were of reagent grade quality, purchased from commercial sources, and used as received.

### Physical Techniques

Attenuated total reflection infrared spectroscopy, performed in a JASCO FT/IR-4000, was employed to record the IR spectra of reaction solutions (400 to 4000 cm^–1^) by dropping a small sample of the solution on the ATR crystal. Gas chromatography (GC) and gas chromatography coupled to mass spectrometry (GC–MS) were performed in gas chromatographs with 25 m capillary columns filled with 1 or 5 wt % phenylsilicone (Shimadzu GC-2025, Agilent GC 6890 N coupled with Agilent MS-5973). ^1^H, ^13^C, and ^19^P nuclear magnetic resonance (NMR) spectra were recorded at room temperature on a 400 MHz spectrometer (Bruker Ascend 400).

### General Procedure for the Isomerization Reaction of **1** with Ru Complex Catalysts

Methyl eugenol **1** (0.2 mL, 1.1 mmol) was charged in a 2 mL vial equipped with a magnetic stirrer, and the corresponding catalyst (0.0001–0.5 mol %) was added directly or dissolved in dichloromethane. The vial was closed with a cap and placed in a steel block at 150 °C under magnetic stirring for a given reaction time. Aliquots of the reaction mixture were taken to follow the reaction over time by GC and NMR.

It should be noted that catalyst stock solutions were prepared when it was required to use very low amounts of catalyst. To prepare the solutions, volumetric flasks were used with dichloromethane as a solvent.

### Isomerization Reaction of **3** and **5** with Ru Complex Catalysts

Terminal alkene (**3** or **5**) was charged in a 2 mL vial equipped with a magnetic stirrer, and the corresponding catalyst (0.005 mol % for **3** and 0.1 mol % for **5**) was added directly or dissolved in dichloromethane. The vial was closed with a cap and placed in a steel block at 150 °C under magnetic stirring for a given reaction time. Aliquots of the reaction mixture were taken to follow the reaction over time by GC and NMR.

### Blank Experiment with Anisole

Anisole (0.5 mL, 4.6 mmol) and RuCl_2_(PPh_3_)_3_ (0.5 mol %, 22 mg) were charged in a 2 mL vial equipped with a magnetic stirrer. The vial was closed with a cap and placed in a steel block oil at 150 °C under magnetic stirring for 3 h. After that, 10% volume of CDCl_3_ was added, and the mixture was analyzed by ^31^P NMR.

### Eyring Plots for the Isomerization Reaction of **1**

Methyl eugenol **1** (0.2 mL, 1.1 mmol) and Grubbs 2nd-generation catalyst (0.2 mol %) were charged in a 2 mL vial equipped with a magnetic stirrer. The vial was closed with a cap, placed in a steel block at the corresponding reaction temperature (150, 175, or 200 °C) under magnetic stirring, and maintained during the reaction time. Aliquots of the reaction mixture were taken to follow the reaction over time by GC and NMR.

### Eyring Plots for the Metathesis Reaction of **2**

Methyl isoeugenol **2** (0.2 mL, 1.1 mmol) and Grubbs 2nd-generation catalyst (0.2 mol %) were charged in a 2 mL vial equipped with a magnetic stirrer. The vial was closed with a cap, placed in a steel block at the corresponding reaction temperature (50, 75, or 90 °C) under magnetic stirring, and maintained during the reaction time. Aliquots of the reaction mixture were taken to follow the reaction over time by GC and NMR.

### Poisoning Experiments (Addition from Start)

Methyl eugenol **1** (1 mL, 5.8 mmol), 0.05 mol % of the catalyst, RuCl_2_(PPh_3_)_3_ (2.79 mg) or Ru_3_(CO)_12_ (1.86 mg), and the corresponding poisoner (1,10-phenanthroline, KSCN, or NaCN) were charged in a 2 mL vial equipped with a magnetic stirrer. The vial was closed with a cap, placed in a steel block at 150 °C under magnet stirring, and maintained during the reaction time. Aliquots of the reaction mixture were taken to follow the reaction over time by GC and NMR.

### Poisoning Experiments (Addition at 10 min)

Methyl eugenol **1** (1 mL, 5.8 mmol) was charged in a 2 mL vial equipped with a magnetic stirrer, and the corresponding catalyst (0.005 mol %, 100 μL of a catalyst stock solution in dichloromethane) was added. The vial was closed with a cap and placed in a steel block at 150 °C under magnetic stirring. After 10 min, 0.5, 1, 2, or 5 equiv of the corresponding poisoner (1,10-phenanthroline or KSCN) were added. Aliquots of the reaction were taken to follow the reaction over time by GC and NMR.

It should be noted that stock solutions were prepared to add the catalyst and poisoner since the amounts used are too small to be weighed. To prepare the solutions, volumetric flasks were used with dichloromethane (catalysts) or methanol (poisoners) as a solvent.

### Reaction Order Experiments of **1** with RuCl_2_(PPh_3_)_3_ and 1,2-Dimethoxybenzene

Methyl eugenol **1** (4.3–1.5 mmol) and a solution of RuCl_2_(PPh_3_)_3_ in dichloromethane (0.001 mol %) and 1,2–dimethoxybenzene (0.25–0.75 mL) were charged in a 2 mL vial equipped with a magnetic stirrer. The vial was closed with a cap and placed in a pre-heated bath oil at 150 °C under magnetic stirring for a given reaction time. Aliquots of the reaction mixture were taken to follow the reaction over time by GC and NMR.

### Isomerization Reaction of **1** under an Inert Atmosphere

In a 10 mL round-bottomed flask equipped with a magnetic stir bar, methyl eugenol **1** (2 mL, 11 mmol) and RuCl_2_(PPh_3_)_3_ (0.001 mol %) were added. The round-bottomed flask was placed in a pre-heated bath oil at 150 °C under magnetic stirring and with or without a N_2_ atmosphere for a given reaction time. Aliquots of the reaction mixture were taken to follow the reaction over time by GC and NMR.

### Cyclic Voltammetry

Electrochemical experiments were performed in 100 ppm solutions of the Ru complexes in neat alkene after adding an equal volume of 0.10 M Hex_4_NPF_6_/MeCN acting as an electrolyte. No deaeration was performed in order to reproduce the experimental conditions of catalytic experiments. Measurements were carried out at 25 °C. A conventional three-electrode electrochemical cell was used with a Pt wire pseudo-reference electrode, a glassy carbon working electrode (GCE, BAS MF 2012, geometrical area 0.071 cm^2^), and a platinum mesh auxiliary electrode. The potentials were calibrated relative to the ferrocenium/ferrocene (Fc^+^/Fc) couple after addition of ferrocene until 0.5 mM concentration to the problem solutions. Cyclic voltammetry (CV) and square wave voltammetry were used as detection modes.

### Orbitrap Measurements

The flow injection-HRMS consisted of an injection and pump system and a single mass spectrometer Orbitrap Thermo Fisher Scientific (Exactive) using an electrospray interface (ESI) (HESI-II, Thermo Fisher Scientific) in positive or negative mode. The injector was directly connected to the source, and 10 μL of the sample was injected into the flow-injection solvent consisting of an aqueous solution of 0.1% formic acid and methanol (1:1). The flow rate remained at 0.20 mL min^–1^ over 5 min. The ESI parameters were as follows: spray voltage, 4 kV; sheath gas (N_2_, >95%), 35 (non-dimensional); auxiliary gas (N_2_, >95%), 10 (non-dimensional); skimmer voltage, 18 V; capillary voltage, 35 V; tube lens voltage, 95 V; heater temperature, 305 °C; capillary temperature, 300 °C. The mass spectra were acquired employing two alternating acquisition functions: (1) full MS, ESI+, without fragmentation [higher collisional dissociation (HCD) collision cell was switched off], mass resolving power = 25,000 FWHM (full width at half-maximum); scan time = 0.25 s; (2) all-ion fragmentation (AIF), ESI+, with fragmentation (HCD on, collision energy = 30 eV), and mass resolving power = 10,000 FWHM; scan time = 0.10 s. The mass range was 150.0–1500.0 *m*/*z*. The chromatograms were processed using Xcalibur version 2.2, with Qualbrowser (Thermo Fisher Scientific).

### Computational Calculations

Molecular models were designed by coupling ruthenium to 1-butene as an alkene model. Resulting geometries were fully optimized by using the dispersion-corrected version of density functional theory (DFT-D3) that implements the Grimme’s pair-wise additive method^9^ in the B3LYP functional (B3LYP-D3).^10^ The Def2SVP basis set was used for all atoms except Ru, which was mimicked with the corresponding relative effective core potential (Def2-ECP) version,^11,12^ a combination that have been successfully used in simulations of organometallic compounds.^13^ Vibrational modes were assessed for all optimized geometries to confirm their nature of minima (all frequencies were real) or transition states (one single imaginary frequency associated to the reaction coordinate). Ligand–metal interaction energies were computed with the counterpoise scheme, a method that accounts for the superposition error computed.^14^ All *ab initio* simulations were performed with the Gaussian 16 suite of programs.^15^

## Results and Discussion

### Formation of the Catalytically Active Ru Species

**Figure 1:**
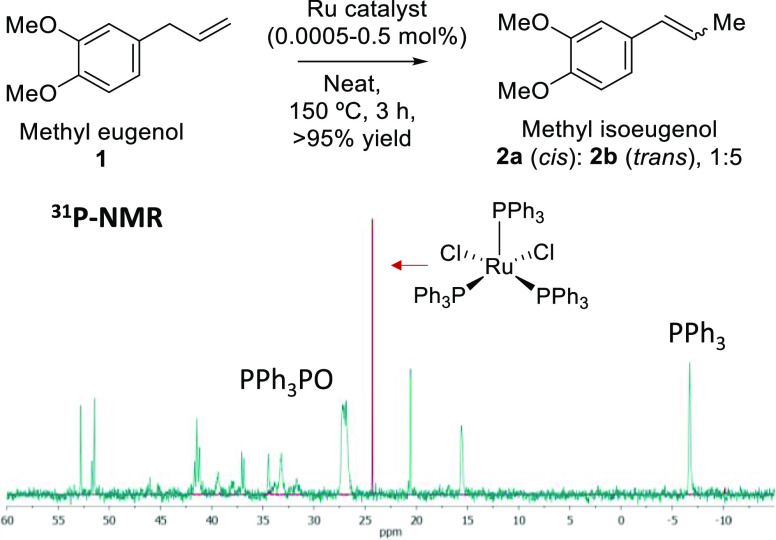
Top: Isomerization reaction of **1** to **2** with 0.0005 (5 ppm)–0.5 mol % Ru complexes or salts at 150 °C. Bottom: ^31^P NMR spectra of the starting RuCl_2_(PPh)_3_ complex (0.5 mol % for better visualization) before (red line) and after 2 h reaction time (blue line) under the indicated reaction conditions. A 10% volume of CDCl_3_ was added for shimming.

In our previous work, we have shown that ppm amounts of virtually any Ru salt and complex tested catalyze the isomerization of methyl eugenol **1** to methyl isoeugenol **2** (*cis*/*trans* mixture).^5^ Kinetic experiments for the reaction of **1** show a clear induction time for the most active complexes Ru(methallyl)_2_(COD), Ru_3_(CO)_12_, and RuCl_2_(PPh_3_)_3_, when using just 1 ppm of Ru (Figure S1). Figure 1 shows the ^31^P NMR spectrum of the reaction mixture when RuCl_2_(PPh_3_)_3_ is used as a Ru pre-catalyst. The result shows that the PPh_3_ ligands completely come off from the complex, and this occurs in a wide range of catalyst concentrations (0.0005–0.5 mol %). A blank experiment in anisole shows that the complex is stable when heated at 150 °C for 3 h (Figure S2). Anisole is a very similar molecule to **1** but without the alkene functionality and allows one to follow the Ru complex by in situ NMR, without any external perturbation. These results suggest that the starting Ru source is transformed into catalytically active Ru species in the hot terminal alkene during the first minutes of reaction, which fits with the observation of an induction time for the isomerization reaction.

**Figure 2:**
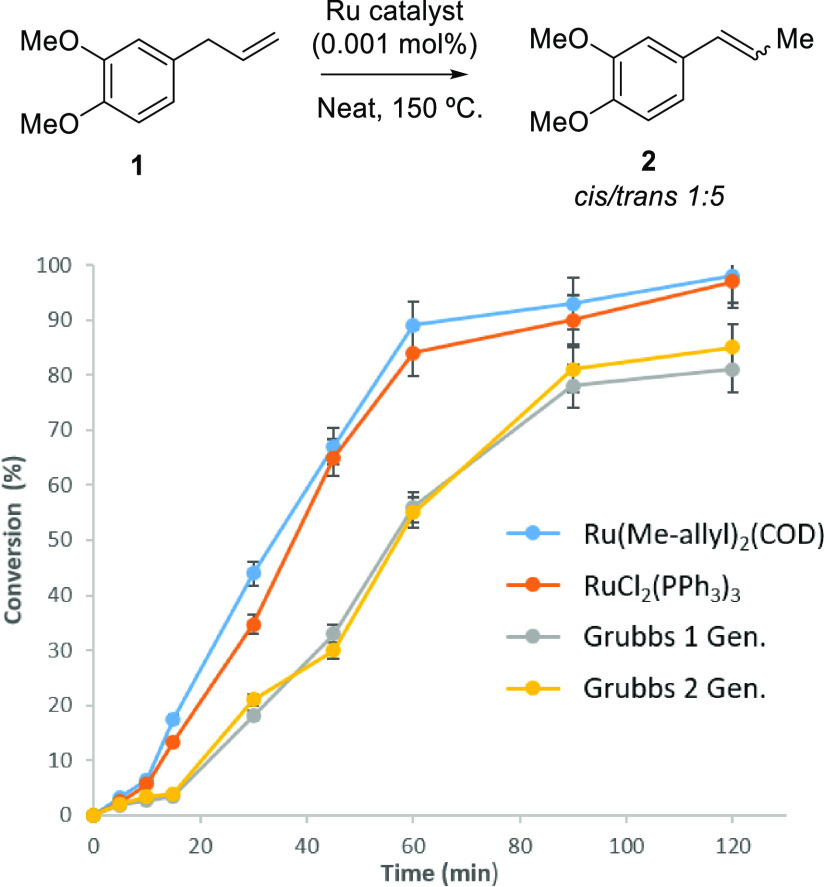
Kinetics for the isomerization reaction of **1** to **2** with 0.001 mol % (10 ppm) of Ru complexes at 150 °C. The results were obtained by GC and confirmed by ^1^H, ^13^C, and ^31^P NMR. Initial rates are calculated from the linear part of the kinetic curve with a maximum slope (after the induction time). Error bars represent a 5% uncertainty.

We then tested 1st- and 2nd-generation Grubbs catalysts as a Ru source of isomerization catalyst in order to follow not only the potential decomposition of the complexes during reaction^16^ but also the competitive isomerization/metathesis reactions. In this way, we can double-check the structure–activity relationship of the catalytic Ru species. Grubbs catalysts are known to promote alkene isomerization reactions, but the active species involved in this undesired reaction for metathesis catalysts are still unknown. The new catalytic results are shown in Figure 2.

It can be seen above that all the Ru complexes show a ∼10 min induction time after which the isomerization reaction starts, showing a high catalytic activity in all cases. While the catalytic activity of both Grubbs catalysts is lower than that of other Ru complexes for the isomerization of **1** to **2**, the catalytic activity becomes very similar for other more-demanding substrates such as allyl anisole derivatives (**3** to **4**, Figure S3) and pentafluorobenzene allyl derivatives (**5** to **6**, Figure S4). These results suggest the formation of the same Ru active species from the four Ru complexes tested.

The lack of any metathesis product during the isomerization reaction of methyl eugenol **1** to methyl isoeugenol **2** with a Grubbs catalyst could be ascribed to a higher activation energy for the former. However, Eyring plots for the isomerization reaction of **1** to **2** (Figure S5) and the metathesis reaction of **2** to stilbene derivative **7** (Figure S6, **2** does not isomerize but only performs the metathesis reaction), which run under exactly the same reaction conditions, show that the calculated activation energies for the isomerization and metathesis reactions are 5.3 and 6.3 kcal·mol^–1^, respectively.^17^ A just 1 kcal difference does not explain the lack of metathesis reaction when using the Grubbs catalysts, and the rapid decomposition of the Ru complexes under the isomerization reaction conditions seems the more plausible explanation.^18,19^ Indeed, ^31^P NMR spectra of the reaction crudes clearly show that the 2nd-generation Grubbs catalyst completely decomposes during the isomerization reaction of **1**, as assessed by the quantitative formation of the corresponding phosphine oxide PO(cyclohexyl)_3_. However, the Ru catalyst mainly preserves the original signals during the metathesis reaction of **2** (Figure S7). In other words, the Grubbs catalysts decompose in the presence of the terminal alkene **1** under heating but do not decompose in the presence of internal alkene **2** under identical reaction conditions. Incidentally, these results also explain the much higher reactivity of **2** with respect to **1** for the metathesis reaction.

### Characterization of the Catalytic Ru Species

In order to further explore the formation of “ligand-free” Ru species in the reaction solution, high-angle annular dark-field scanning transmission electron microscopy (HAADF*–*STEM) measurements of the liquid reaction mixture were performed. 1-Decene **8** was used as a reactant^5^ since all the alkenes **1–7** previously tested are too heavy for volatilization off the microscopy grid after the reaction. The results (Figure S8, circled areas highlight the heavier metal atoms detected with the instrumentation) infer that single Ru atoms (oxidation state still unknown) are the only species present in the mixture when using a 0.01 mol % of RuCl_2_(PPh_3_)_3_ as a pre-catalyst, at 150 °C for 2 h, which is perhaps surprising considering that a 0.01 mol % of Ru should generate agglomerated species under the heating reaction conditions here employed.

**Figure 3:**
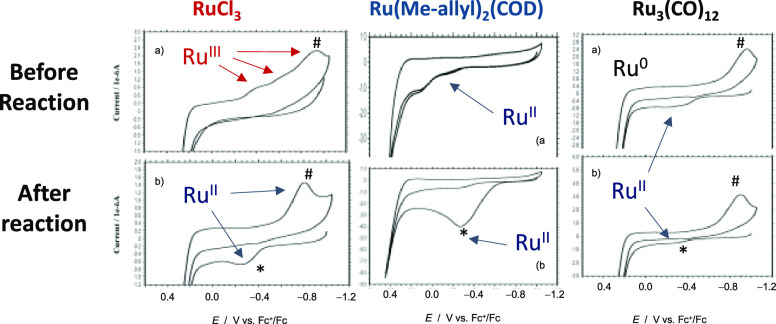
Cyclic voltammogram at the glassy carbon electrode for the isomerization of **1** to **2** in 1:1 v/v solutions with 0.01 mol % (100 ppm) of RuCl_3_, Ru(methylallyl)_2_(COD), and Ru_3_(CO)_12_ in **1** plus 0.10 M Hex_4_NPF_6_/MeCN. Potential scan initiated at −1.0 V vs Fc+/Fc in the positive direction; potential scan rate 50 mV·s^–1^. The figure compares the voltammograms before (a, upper file) and after the reaction (b, lower file). The more informative signals are marked with arrows. Peaks marked with * and # denote the Ru(II) to Ru(III) oxidation and the reduction of Ru(II)–MeCN species generated during the electrochemical process, respectively.

We then used CV as an in situ technique to assess the oxidation state of the Ru species in solution. Figure 3 shows CV measurements with RuCl_3_, Ru(methallyl)_2_(COD), and Ru_3_(CO)_12_ as pre-catalysts for the isomerization of **1** to **2**. RuCl_2_(PPh_3_)_3_ and Grubbs catalysts could not be measured by the potential oxidation of the phosphine ligands. Notice that the voltammetric experimental conditions employed here (see Experimental Section) are reasonable comparable with the synthetic reaction conditions. The results indicate that, regardless of the initial oxidation state of the metal, Ru(II) to Ru(III) oxidation signals are prevalent after the isomerization reaction. In all cases, the voltammograms collapse to a quite similar profile consisting of a unique, well-defined anodic wave at −0.30 V in the initial anodic scan (indicated with an asterisk), which is a blueprint of Ru(II) *per*alkene complexes,^20^ formed after the reaction with isoeugenol **1**. These Ru(II) *per*alkene complexes are achieved in voltammetric experiments regardless of the initial Ru source, either by the oxidation of Ru(0) or by the reduction of Ru(III) under the heating reaction conditions. For instance, upon starting the measurement of the reaction mixture with a potential at −1.0 V vs Fc+/Fc in the positive direction, the reaction containing RuCl_3_ shows no oxidation signals but display three overlapping reduction peaks between −0.4 and −1.0 V in the subsequent cathodic scan. These signals can be attributed to the stepwise reduction of Ru(III) to Ru(II) and Ru(0),^21^ accompanied by the partial complex dissociation and formation of Ru(II)(MeCN)_*n*_ complexes, which in turn reduced to Ru(0).^22^ In a similar way, the voltammetry with the Ru(methallyl)_2_(COD) complex shows two overlapping anodic waves between 0.0 and 0.4 V, which can be attributed to the irreversible Ru(II) to Ru(III) oxidation, presumably also involving some MeCN-coordinated form. Ru_3_(CO)_12_ also evolves to Ru(II) during the reaction, although showing an ill-defined anodic wave at ca. −0.2 V preceding a prominent anodic current ca. 0.5 V. However, in the subsequent negative-going potential scan, a cathodic peak appears at −0.6 V. This signal is quite similar to the third cathodic wave recorded for Ru(III) and can be assigned to the reduction of the Ru(II)(MeCN)_*n*_ species, previously generated in the anodic scan.

To further visualize the Ru–alkene complex, an Orbitrap analyzer with flow injection-mass spectrometry (HPLC–Orbitrap MS) was employed.^5^ This instrumentation allows us to detect ppm of organic and organometallic compounds, and the analysis of the reaction mixture with Ru(methallyl)_2_(COD) (Figure S9, top, 300 ppm for a better visualization) shows the disappearance of the Ru precursor and the formation of a Ru *per*alkene complex, with the expected isotopic distribution for one Ru atom. The simulated spectra (Figure S9, bottom) fit well the experimental ones.

**Figure 4:**
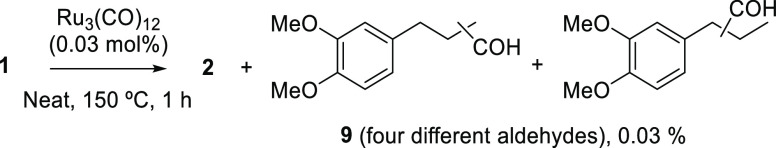
Byproducts found during the isomerization reaction of **1** to **2** with 0.03 mol % (300 ppm) of Ru_3_(CO)_12_ at 150 °C. The results were obtained by GC–MS analyses.

In order to further visualize the disappearance of the Ru ligands during the reaction, the isomerization of **1** to **2** was followed by Fourier-transformed infrared spectroscopy (FT-IR), employing Ru_3_(CO)_12_ as a catalyst. The use of this Ru precursor here is well justified since the CO ligands are the most sensitive to detect by this technique and, besides, the corresponding peaks appear in a clear area of the spectrum (2100–2200 cm^–1^). The results (Figure S10, 300 ppm of the Ru precursor for a better visualization) show the nearly complete disappearance of the CO ligands, which supports that the ligands come off during the reaction. These results are confirmed after the analysis of the reaction mixture by GC–MS (Figure S11) since Figure 4 shows the quantitative formation of the aldehyde products **9**, which come from the Ru-catalyzed hydroformylation reaction of the alkenes **1** and **2** with the CO ligands of the catalysts. These results reveal the release of the Ru ligands during the reaction, the catalytic activity of the Ru(II) atom after ligand removal (considering that Ru(0) precursors are rapidly oxidized under the reaction conditions, see also below), and the fate of CO during the reaction. Notice that the amount of aldehyde byproducts under optimized reaction conditions, i.e., <10 ppm of Ru_3_(CO)_12_, is extremely low, and that this reactivity avoids the formation of extremely toxic-free CO during the reaction.

**Figure 5:**
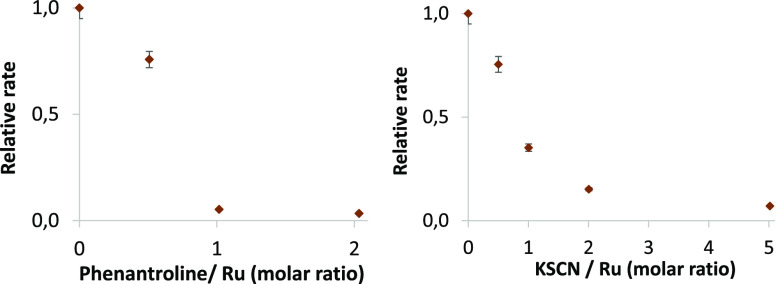
Inhibition plots (relative reaction rate–amount of poison) for the isomerization reaction of **1** to **2** with 0.005 mol % (500 ppm) of RuCl_2_(PPh_3_)_3_ at 150 °C with either 1,10-phenanthroline (left) or KSCN (right). The results were obtained by GC–MS analyses. Initial rates are calculated from the linear part of the kinetic curve with a maximum slope (after the induction time). Error bars represent a 5% uncertainty.

Catalyst poisoning experiments with three different anions/bases, i.e., CN^–^, SCN^–^, and 1,10-phenanthroline,^23^ were carried out in order to assess that the oxidation state is Ru(II), either starting from RuCl_2_(PPh_3_)_3_ or from Ru_3_(CO)_12_. The polar compounds in small amounts are soluble, at least visually, under the heating reaction conditions employed. The results (Figures S12–S14) show that the addition of most of these Lewis bases completely inhibits the isomerization reaction of **1** when added from the beginning of the reaction, with both Ru catalysts (Figure S12). However, when added at 30% conversion, the poisoning is partial and allows us to estimate the number of Ru atoms in the true catalyst (Figures S13–S14). Figure 5 shows the plots correlating the reaction rate (relative to the rate without any poison in reaction)–amount of poison, which indicates that either two molecules of 1,10-phenanthroline or four molecules of KSCN are enough to completely stop the catalytic activity, which correspond to a single tetracoordinated Ru atom. FT-IR analyses were performed to detect the formation of the corresponding Ru(II)–Lewis base adduct during the reaction (Figure S15); however, it is difficult to observe these adducts in the presence of the reactant. In any case, the inhibition of the reaction by different anions/bases support the in situ formation and catalytic activity of Ru(II)–*per*alkenes in the isomerization reaction, regardless of the starting Ru source.

**Figure 6:**
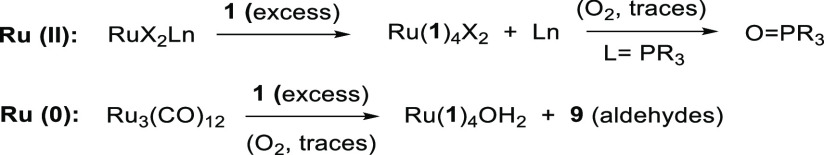
Equations depicting the plausible formation of Ru(II) *per*alkene complexes from either Ru(II) or Ru(0) precursors.

The combined kinetics, ^31^P NMR, HAADF*–*STEM, CV, MS, FT–IR, and reactivity results shown above support the formation of Ru(II) *per*alkene complexes in solution, during the reaction, as the catalytically active species for the isomerization reaction of terminal alkenes. Figure 6 shows a plausible equation for the formation of the active Ru(II) species, either from Ru(II) or Ru(0) precursors. The equations show that the anions must stay with the Ru(II) atoms, plausibly as charge counterbalancing anions out of the coordination shell, which may be fully occupied by alkenes (since they are in extremely high excess). Otherwise, the Ru precursor anions should have an impact on the catalytic activity, which is not the case.^5^ The potential action of O_2_ is also considered, despite the fact that the isomerization reaction proceeds similarly under an open and inert atmosphere (Figure S16), since traces of O_2_ (or alternatively a redox reaction with **1**) could easily oxidize the bare and instable Ru(0) atoms (see CV experiments above). Please notice that the study of the Ru active species is severely limited by the extremely low concentration of catalytic Ru (ppm), and many other characterization techniques could not be applied.

### Plausible Formation of the Ru Active Species by a Finke–Watzky Mechanism

The above results clearly indicate that the catalytically active Ru species are formed after an induction period. The fact that most of the Ru precursors are able to form these species when heating at >100 °C in the neat terminal alkene, in combination with the above-commented findings, indicates that Ru(II) *per*alkene complexes are the plausible catalytic species (Tables S1–S5). However, the lack of any metathesis product when starting from Grubbs catalysts suggests that the formation of the Ru(II) *per*alkene complexes is fast. Thus, other process is responsible for the induction time.

Computational calculations were carried out to assess the relative stability of the different Ru complexes and the postulated Ru(II) *per*alkene catalyst (Tables S6–S7). As for the Ru(methallyl)_2_(COD) complex is concerned, theory predicts that the COD (COD stands for 1,5-cyclooctadiene) moiety is anchored to the metallic center with an interaction of −90 kcal/mol, while each CO binds to Ru with an energy of −48 kcal/mol in the Ru_3_(CO)_12_ counterpart. The larger interaction (more negative value) in the former pre-catalyst complex arises from the double contact with the ligand. A significant larger interaction energy is predicted for the Ru(II) *per*alkene complex, where terminal alkenes (1-butene) enter into the metallic sphere with an energy of −64 kcal/mol. The larger affinity toward the terminal alkene suggests that other chemical events are producing the induction time observed during the formation of the catalytically active Ru species, i.e., the start of the isomerization reaction, since the formation of a Ru(II) *per*alkene complex under the heating reaction conditions must be extremely fast.

**Figure 7:**
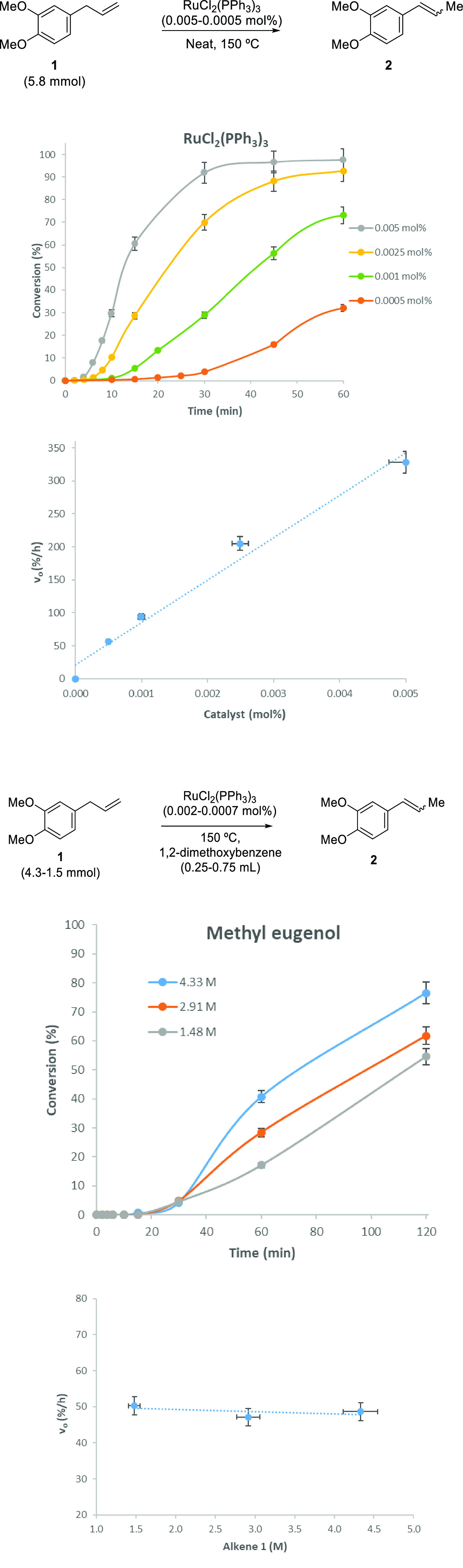
Reaction orders for the isomerization of methyl eugenol **1** to methyl isoeugenol **2** catalyzed by 5–50 ppm of Ru_3_Cl_2_(PPh_3_)_2_ at 150 °C. The reaction order experiments for the alkene were carried out by keeping the total volume of the reaction constant at 1 mL, adding a 2.9 × 10^–5^ mmol of catalyst in a stock solution to each reaction (7–20 ppm), and varying the amount of alkene and solvent (1,2-dimethoxybenzene). Top: Ru catalyst. Bottom: alkene **1**. The reaction order for Ru_3_Cl_2_(PPh_3_)_2_ is 1, and the reaction order for alkene **1** is 0. Please notice that the same order is obtained for Ru(methyallyl)_2_(COD) in a previous work.^5^ Initial rates are calculated either from the linear part of the kinetic curve with a maximum slope (after the induction time) or by the *k*_1_ value to give similar values (see Tables S1–S5). Error bars represent a 5% uncertainty.

The sigmoidal curves observed for the isomerization reaction are compatible with a Finke–Watzky mechanism for catalyst formation, as shown in Figure 7 (see also Tables S1–S5). Besides, the rate law obtained for the isomerization reaction of **1**, on the basis of both *k*_1_ and initial rates (within a ∼5% error), is *v*_0_ = *k*_exp_[Ru], regardless of the initial Ru source; thus, the formation of the catalyst is the only parameter controlling the reaction rate, which is in accordance with a Finke–Watzky autocatalytic catalyst formation mechanism.

This mechanism applies to the exponential formation of the catalytically active species from a metal precursor, following eq 1.
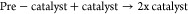
1

These steps can be described in a pseudo-elementary step (PEStep) model, as shown in eq 2. Here, the reaction acts as a catalytic reporter reaction (CRR),^7^ allowing us to follow the formation of the true catalyst. The example below is for 5 ppm of Ru, taking into account that there is not any solvent nor additives in the reaction.
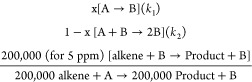
2

**Figure 8:**
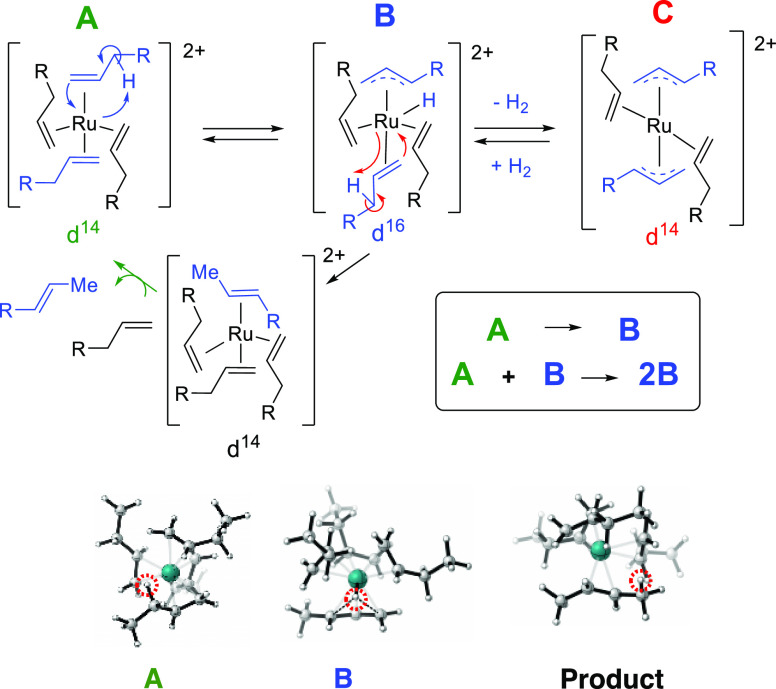
Proposed Finke–Watzky mechanism (A → B, then A + B → 2B) for the formation of the catalytically active Ru species during the induction period of the isomerization of terminal alkenes (top). Computational calculations: DFT structures for **A**, **B** and the product. Transferred hydrogens upon the isomerization process are circled in red. Color scheme: gray, C atoms; green, Ru atoms; white, H atoms (bottom).

For this mechanism to operate, small amounts of the true Ru catalyst must be generated during the induction time and react with a Ru pre-catalyst, to form more Ru catalysts, in an exponential way. Figure 8 shows our proposal here for the generation of these catalytically active Ru species. The corresponding *k*_1_ and *k*_2_ values for different specific reactions are calculated in the Supporting Information (Tables S1–S5).

The decomposition of the Ru precursor in the neat terminal alkene leads to a rapid formation of a 14-electron complex Ru(II) *per*alkene complex (**A**), which is then transformed to the corresponding 16-electron complex **B** after Ru–H insertion. The performed simulations show that this process is associated to a low energetic barrier (4 kcal/mol). In addition, **A** and **B** are mainly isoenergetic, with a difference of just 0.3 kcal/mol. As sketched in Figure S11, the **B** complex can still evolve to the 14-electron complex **C** after losing H_2_. Our calculations demonstrate that only complex B leads to a minimum in the potential energy surface associated to the hydrogen transference, thus only **B** generates the product. This is the consequence of the optimal orientation for the transferred hydrogen in **B**, which is located at the middle point between the involved carbons (see Figure 8, bottom panel). The hydrogen atom is subsequently transferred from the metallic center to the terminal carbon via a barrierless reaction, which eventually leads to the internal alkene.^24,25^ It is remarkable that the terminal alkene is associated to a higher intense interaction (−37 kcal/mol) comparted to its internal counterpart (−64 kcal/mol, see above). The fact that internal alkenes bind less strongly than terminal alkenes to Ru most probably comes from combined metal–alkene highest occupied/lowest unoccupied molecular orbital (HOMO–LUMO) interactions, thus electronic effects rather than steric effects.^26^ The numeric outputs confirm that a new catalytic cycle might start by replacing the resulting internal isomer with a new incoming terminal alkene, which in turn regenerates complex **A**.^27−29^ In this way, the Finke–Watzky autocatalytic catalyst formation mechanism can operate (see eq 1).

We have also performed kinetic experiments to represent the relationship between the amount of the catalyst and induction time for three different Ru catalytic precursors. The results (Figure S17) show an exponential correlation between the induction time and amount of the catalyst, regardless of the Ru precursor, which nicely supports the formation of the catalytically active Ru species by the proposed A + B = 2B equation, up to a certain saturation limit for Ru.

**Figure 9:**
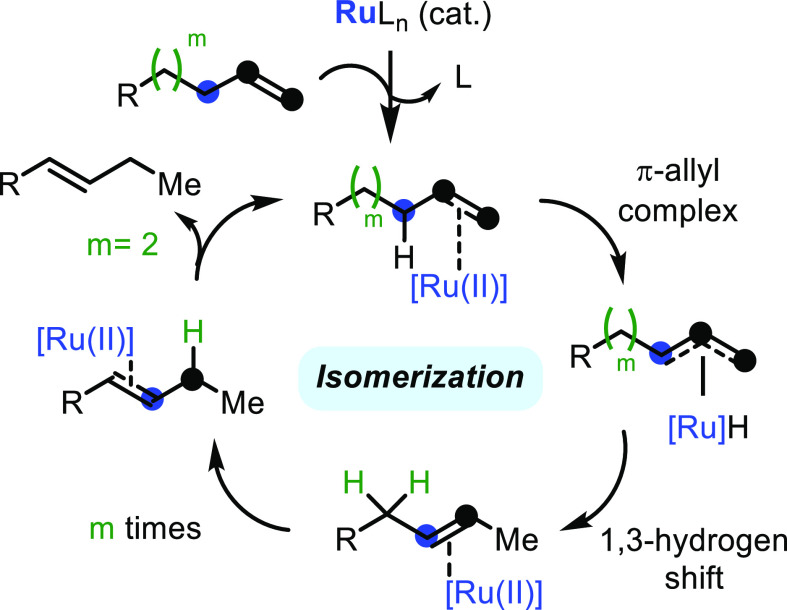
Plausible mechanism for the isomerization reaction of the terminal catalyzed by ppm of Ru(II).^5^

With the pseudo-elementary steps in hand,^30^ we propose that the isomerization of **1** proceeds through a Finke–Watzky mechanism for catalyst formation. Sub-nanometric species or Ru nanoparticles have not been observed with any precursor and are in principle discarded.^31,32^ The role of the counteranions seems to be of low relevance, and the potential catalytic activity of in situ formed Brönsted acids such as HCl is discarded on the basis of kinetic experiments (Figure S18). With all these data in hand, we propose a mechanism for the isomerization reaction of the terminal catalyzed by ppm of Ru, as shown in Figure 9.^5^ This mechanism is consistent with previous works on Ru-catalyzed isomerization reactions of alkenes.^33,34^

## Conclusions

The plausible catalytically active Ru species for the isomerization of terminal alkenes with ppm of many Ru sources are Ru(II) *per*alkene complexes, formed in situ during the reaction and present as species in the catalytic cycle. These species may also be behind the isomerization processes found during Ru-catalyzed alkene metathesis reactions. Although it is true that we have not completely proved the nature of the Ru catalysts, we have disproved many potential catalytic species, such as Ru big clusters and nanoparticles. The energetically favored formation of the Ru(II) *per*alkene complex under heating conditions in neat terminal alkenes, regardless of the starting Ru source, is the thermodynamic force which boosts the reaction with such low amounts of catalytic metals. A Finke–Watzky mechanism for catalyst formation enables the rapid formation of the 16-electron complex Ru(II)–H catalytic species in a liquid phase. These results open new ways to catalyze the isomerization of terminal to internal alkenes, which are of high interest in a variety of chemical processes.^35−38^

## Supporting Information Available

Additional experimental data and compound characterization and NMR copies (PDF)
