# Supplementary material for: Evidence for Ruthenium(II) Peralkene Complexes as Catalytic Species during the Isomerization of Terminal Alkenes in Solution
Source: Inorg Chem. 2023 Jul 3;62(28):10984–92. doi: 10.1021/acs.inorgchem.3c00967 (PMC10354743; doi:10.1021/acs.inorgchem.3c00967)
Supplement: Supplementary file 1 — ic3c00967_si_001.pdf [file ic3c00967_si_001.pdf]

SUPPORTING INFORMATION

Evidence for ruthenium(II) peralkene complexes as catalytic species during the isomerization of terminal alkenes in solution

Sergio Sanz–Navarro,[a] Jordi Ballesteros–Soberanas,[a] Aarón Martínez–Castelló,[b] Antonio Doménech–Carbó,[c] Juan Carlos Hernández–Garrido,[d] Jose Pedro Cerón–Carrasco,[e] Marta Mon[a] and Antonio Leyva–Pérez.[a]

[a] Instituto de Tecnología Química. Universitat Politècnica de València–Consejo Superior de Investigaciones Científicas. Avda. de los Naranjos s/n, 46022, Valencia, Spain.

[b] Zschimmer & Schwarz Spain, CTRA. CV–20, KM. 3.200. APDO. 118, 12540, Villareal, Spain

[c] Departament de Química Analítica, Universitat de Valencia, Dr Moliner, 50, 46100, Burjassot, Valencia, Spain.

[d] Departamento de Ciencia de los Materiales e Ingeniería Metalúrgica y Química Inorgánica, Facultad de Ciencias, Universidad de Cádiz, Campus Universitario Puerto Real, 11510 Puerto Real, Cádiz, Spain.

[e] Centro Universitario de la Defensa, Universidad Politécnica de Cartagena. Base Aérea de San Javier. C/ Coronel López Peña S/N, Santiago de La Ribera, 30720, Murcia, Spain.

Table of contents

Supporting Tables (Tables S1 – S7) p. S2-S10

Supporting Figures (Figures S1 – S18) p. S11-S28

Compound characterization p. S29-S30

NMR copies p. S31-S36

Supporting Tables

Table S1. Parameters for the kinetics of **1** with different catalysts in Figure 2.

	catalyst (mol%)	$k_{1\,curve}$ (min^{-1})	$k_{2\,curve}$ ($min^{-1}\cdot M^{-1}$)	correction factor	k_2 ($min^{-1}\cdot M^{-1}$)	v_0 (%/min)
**Ru(Me-allyl)$_2$(COD)**	0.001	2.0733	0.0100	100000	1000.0	1.73
**$RuCl_2(PPh_3)_3$**	0.001	1.9189	0.0087	100000	870.0	1.72
**Grubbs 1st Gen.**	0.001	1.0617	0.0022	100000	220.0	1.27
**Grubbs 2nd Gen.**	0.001	1.1149	0.0029	100000	290.0	1.13

Table S2. Parameters for the kinetics of **3** with different catalysts in Figure S3.

	catalyst (mol%)	$k_{1\,curve}$ (min^{-1})	$k_{2\,curve}$ ($min^{-1}\cdot M^{-1}$)	correction factor	k_2 ($min^{-1}\cdot M^{-1}$)	v_0 (%/min)
**Ru(Me-allyl)$_2$(COD)**	0.005	0.5265	0.0007	20000	14.0	0.56
**$RuCl_2(PPh_3)_3$**	0.005	0.4897	0.0006	20000	12.0	0.07
**Grubbs 1st Gen.**	0.005	0.4723	0.0006	20000	12.0	0.39
**Grubbs 2nd Gen.**	0.005	0.4679	0.0006	20000	12.0	0.49

Table S3. Parameters for the kinetics of **5** with different catalysts in Figure S4.

	catalyst (mol%)	$k_{1\,curve}$ (min^{-1})	$k_{2\,curve}$ ($min^{-1}\cdot M^{-1}$)	correction factor	k_2 ($min^{-1}\cdot M^{-1}$)	v_0 (%/min)
**Ru(Me-allyl)$_2$(COD)**	0.1	0.5039	0.0006	1000	0.6	0.51
**$RuCl_2(PPh_3)_3$**	0.1	0.4795	0.0006	1000	0.6	0.47
**Grubbs 1st Gen.**	0.1	0.4348	0.0005	1000	0.5	0.39
**Grubbs 2nd Gen.**	0.1	0.4545	0.0006	1000	0.6	0.41

Table S4. Parameters for the kinetics of **1** with different catalysts in Figure 7.

RuCl$_2$(PPh$_3$)$_3$					
**catalyst (mol%)**	**$k_{1\,curve}$ (min^{-1})**	**$k_{2\,curve}$ (min^{-1}·M^{-1})**	**correction factor**	**k_2 (min^{-1}·M^{-1})**	**v_0 (%/min)**
0.005	4.7275	0.0492	20000	984.0	5.47
0.0025	3.0752	0.0215	40000	860.0	3.42
0.001	1.7247	0.0069	100000	690.0	1.56
0.0005	0.3290	0.0027	200000	540.0	0.94

Table S5. Parameters for the kinetics of **1** with different catalysts in Figure S17.

Ru$_3$(CO)$_{12}$					
**catalyst (mol%)**	**$k_{1\,curve}$ (min^{-1})**	**$k_{2\,curve}$ (min^{-1}·M^{-1})**	**correction factor**	**k_2 (min^{-1}·M^{-1})**	**v_0 (%/min)**
0.0050	4.6788	0.0509	20000	1018.0	5.42
0.0025	3.5460	0.0310	40000	1240.0	3.17
0.0010	2.1656	0.0113	100000	1130.0	1.74
0.0005	1.3662	0.0046	200000	920.0	0.65

Table S6. Computed energies (in kcal/mol) for A, B and product model systems displayed in Figure 8 in the main text.

	A	B	Product
Total electronic energy (hartrees)	-722.749209	-722.748737	-722.772925
Relative energies (kcal/mol)	0.0	0.3	-14.9

Table S7. DFT Cartesian coordinates for A, B and Product model systems displayed in Figure 8 in the main text.

A

C	1.63458800	1.29665200	-0.53996300
C	2.28834200	0.08846600	-0.32534600
C	2.89651700	-0.73175900	-1.44040100
C	4.39355200	-0.43057300	-1.60274200
Ru	0.01319100	-0.14281900	0.18623900
C	-0.42061700	-0.66368500	-1.80318100
C	-1.42754600	-1.26382200	-0.98920100
C	-2.90002200	-0.95394300	-1.17039300
C	-3.48106200	-1.74749900	-2.34989100
C	0.81609000	-2.17572700	0.68651800
C	-0.04555200	-1.65013700	1.67822800
C	0.54056100	-0.65681800	2.64030000
C	-0.25951100	-0.40590800	3.91453400
C	-1.74146000	0.86967700	1.00175600
C	-1.26501300	1.73844300	-0.00402600
C	-0.77950200	3.13884900	0.34803900
C	-0.37202000	3.99574400	-0.85196800
H	1.67156200	2.09418200	0.20465900
H	1.42261600	1.61874500	-1.56224000
H	2.77178700	-0.06189700	0.64918900
H	2.76138200	-1.80901100	-1.24952100
H	2.37622600	-0.51251800	-2.38557300

H	4.83597000	-1.03450900	-2.41186100
H	4.56081800	0.63273800	-1.83961700
H	4.94900000	-0.65469200	-0.67650000
H	0.37396200	-1.28936900	-2.22014900
H	-0.69665100	0.18603400	-2.44084600
H	-1.28567600	-2.32412600	-0.74695700
H	-3.46144900	-1.21354100	-0.25660500
H	-3.06371800	0.11964500	-1.34840200
H	-4.55765400	-1.54684400	-2.47735200
H	-2.97089300	-1.48345300	-3.29058000
H	-3.35681600	-2.83324100	-2.20116300
H	0.46315500	-2.98283900	0.03706000
H	1.89432200	-2.19710000	0.86592200
H	-1.02462500	-2.08421600	1.90399400
H	1.59057500	-0.90394500	2.87777100
H	0.66460400	0.34970900	2.09069600
H	0.14713400	0.43703100	4.49445200
H	-1.31417700	-0.19087200	3.68957400
H	-0.23203900	-1.30242600	4.55399000
H	-2.65721400	0.29294500	0.88305800
H	-1.55469800	1.16991100	2.04054500
H	-1.76001700	1.69602800	-0.98205200
H	-1.60710000	3.65835700	0.86907500
H	0.03903500	3.09625500	1.08810600
H	-0.09493900	5.01591500	-0.54065900

H	0.47959900	3.56992200	-1.40041900
H	-1.20856300	4.08343100	-1.56567500

B

C	-1.37106400	1.08797100	0.99416800
C	-2.17433200	0.74071400	-0.09485100
C	-3.37076600	-0.17821800	0.04176600
C	-4.57608600	0.57257400	0.62771700
Ru	-0.09391900	-0.14511600	-0.43793200
C	-0.48442900	-1.76491900	1.21670900
C	0.84371600	-1.39275900	1.33694900
C	1.37390900	-0.62354800	2.52565200
C	1.83518600	-1.56049800	3.65200600
C	-1.24503900	-1.47461200	-1.74939200
C	0.08240400	-1.98041400	-1.67194800
C	1.18729900	-1.26469600	-2.13568300
C	2.58582600	-1.80540000	-2.00220400
C	2.02382500	0.83997000	-0.45436400
C	1.22805500	1.77170800	0.18141900
C	0.83059100	3.07377900	-0.47513000
C	1.73491400	4.22905200	-0.02221900
H	-1.01119200	2.10990200	1.09584100
H	-1.51013800	0.57379900	1.94872000
H	-2.29576000	1.49584000	-0.87872300
H	-3.65133700	-0.58808700	-0.94086900

H	-3.12318200	-1.03814400	0.68329000
H	-5.45922900	-0.08363500	0.69836400
H	-4.35230700	0.95436500	1.63712300
H	-4.85052200	1.43765400	0.00104900
H	-0.78390100	-2.67470000	0.69321500
H	-1.21167500	-1.41573800	1.95368800
H	1.59835800	-2.00628900	0.82954000
H	2.22594600	0.01226900	2.22972600
H	0.59536200	0.05628500	2.90764700
H	2.24157500	-0.99359200	4.50592600
H	0.99863500	-2.17815400	4.01666400
H	2.62297900	-2.24731900	3.29972100
H	-2.03151300	-2.09443800	-1.31173800
H	-1.55976400	-0.90588400	-2.62848500
H	0.27779500	-2.89111400	-1.09407300
H	1.03665800	-0.54017000	-2.94040600
H	-0.29316800	0.81997900	-1.68665700
H	3.34134800	-1.00625200	-1.95874600
H	2.70245300	-2.43359800	-1.10561600
H	2.83538500	-2.42840900	-2.87992400
H	2.66439700	0.15707700	0.10428500
H	2.31697200	1.02055400	-1.49084200
H	1.21625400	1.77947300	1.27451200
H	0.87548000	2.95910800	-1.56961400
H	-0.21680500	3.32595000	-0.23885000

H	1.43439700	5.17816100	-0.49505600
H	1.68702800	4.36984600	1.07070900
H	2.78784100	4.03613600	-0.28498100

Product

C	0.73042000	0.52417300	-1.85791200
C	1.78260300	0.44404700	-0.92760400
C	2.96140400	-0.49846600	-1.03967700
C	4.13695500	0.15043400	-1.78414800
Ru	-0.02351700	-0.00274400	0.23215700
C	0.19780600	-2.00406200	-0.41038200
C	-1.18461200	-1.63155500	-0.40192100
C	-2.01400700	-1.51099700	-1.66364600
C	-2.57380200	-2.87399900	-2.09815600
C	2.02276200	0.64872300	2.28525400
C	1.19560900	-0.56709700	1.91551300
C	-0.14463300	-0.66186000	2.37405100
C	-0.80053400	-1.95849100	2.78772500
C	-1.94321000	1.03634200	0.85202200
C	-1.51615600	1.65669100	-0.32146600
C	-0.88546900	3.03495000	-0.30021800
C	-1.92843100	4.14986600	-0.47091300
H	0.33723700	1.48619200	-2.18868000
H	0.62455100	-0.27772900	-2.59616700
H	2.04226300	1.39152000	-0.42224000

H	3.29470500	-0.81428500	-0.03698400
H	2.65864100	-1.41495700	-1.56760000
H	4.99544100	-0.53743500	-1.85559800
H	3.84485200	0.44018600	-2.80666600
H	4.48171800	1.06269800	-1.26844500
H	0.58717900	-2.68914400	0.34874700
H	0.69353300	-2.12004100	-1.37863800
H	-1.78548600	-1.98275600	0.44504100
H	-2.85474000	-0.81594800	-1.49258300
H	-1.41444100	-1.08070700	-2.47937800
H	-3.19295100	-2.78330200	-3.00566900
H	-1.75806100	-3.58382900	-2.31196400
H	-3.19981100	-3.31925500	-1.30695400
H	2.95930000	0.71372100	1.71265500
H	2.28341800	0.63131300	3.35968400
H	1.77186800	-1.48962900	1.77941500
H	-0.50086400	0.18722000	2.97117600
H	1.46282900	1.59074700	2.12613700
H	-1.89889800	-1.90385600	2.70799800
H	-0.46131100	-2.81851500	2.19224400
H	-0.56871600	-2.18578000	3.84435600
H	-2.72955300	0.27797500	0.83989400
H	-1.80814000	1.54343000	1.81315300
H	-1.97243900	1.36593000	-1.27218500
H	-0.34713300	3.18387600	0.65374100

H	-0.12996200	3.12859700	-1.09888100
H	-1.45584000	5.14574400	-0.46939300
H	-2.47209200	4.03553800	-1.42325500
H	-2.67259300	4.12102600	0.34102200

Supporting Figures

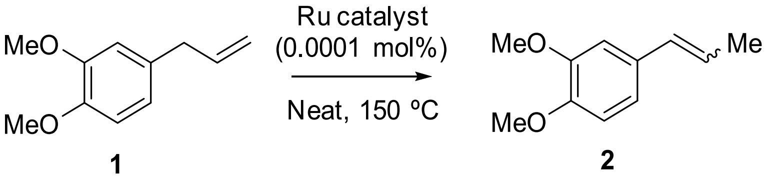

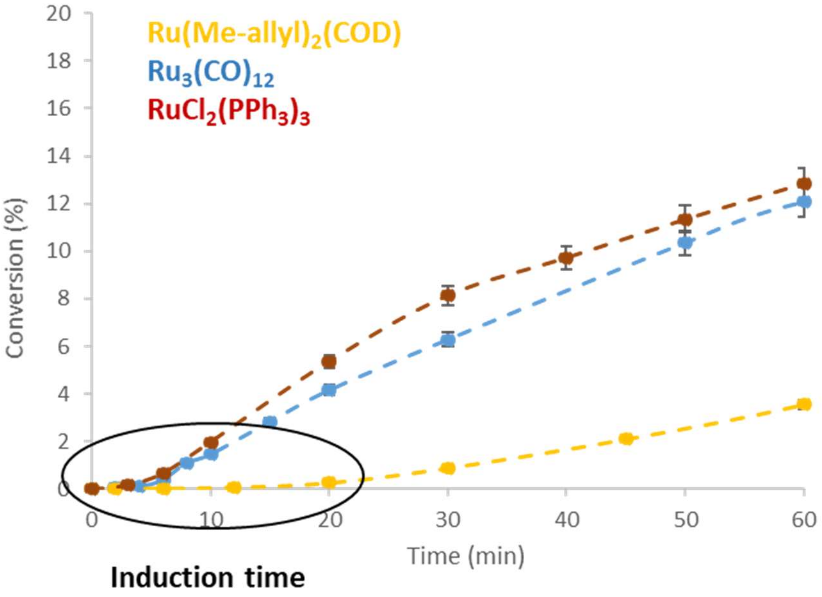

Figure S1. Kinetics for the isomerization of methyl eugenol **1** to methyl isoeugenol **2** catalyzed by 1 ppm of different Ru complexes at 150 ºC.

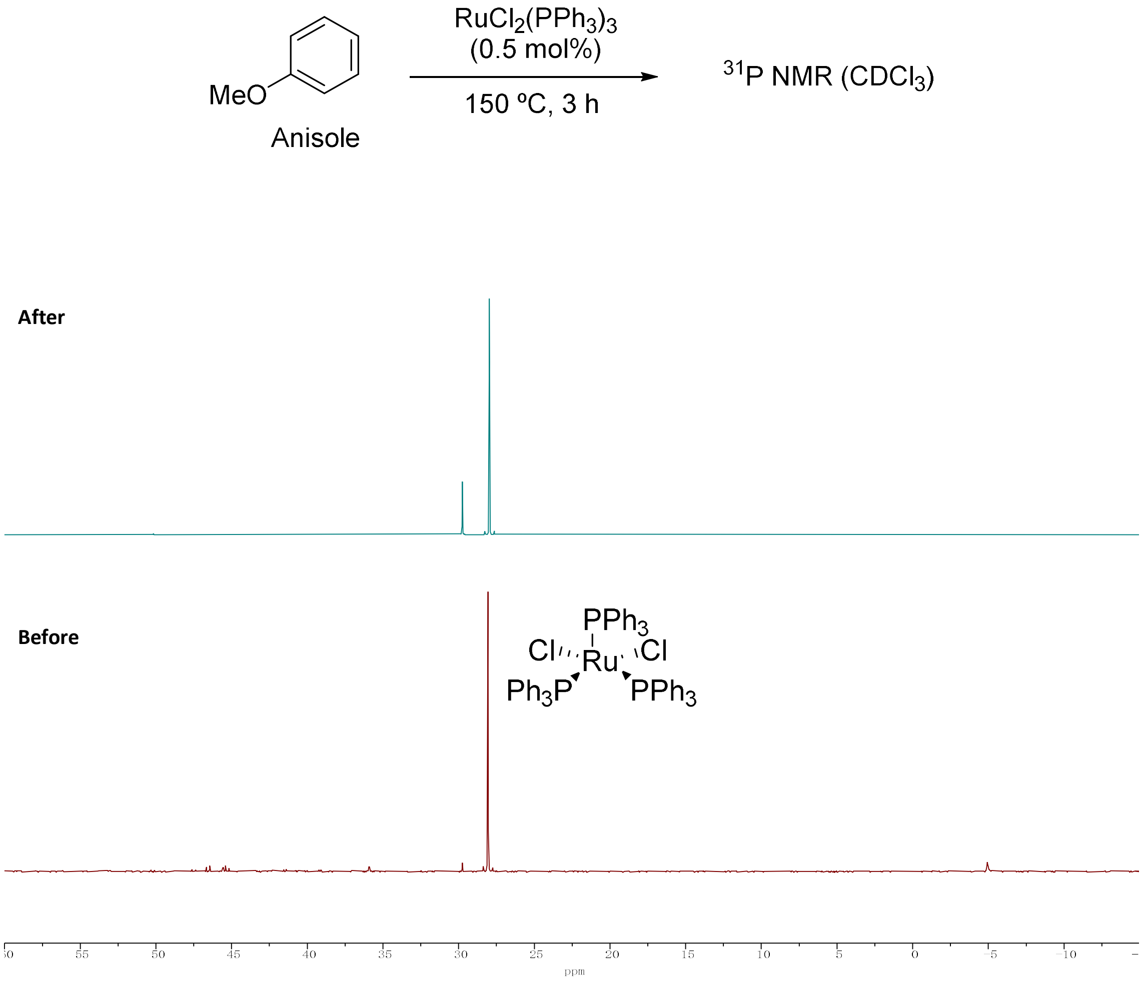

Figure S2. ^{31}P–NMR spectra of the starting $RuCl_2(PPh)_3$ complex (0.5 mol%) before and after heating at 150 ºC for 3 h in anisole. A 10 % volume of $CDCl_3$ was added for shimming.

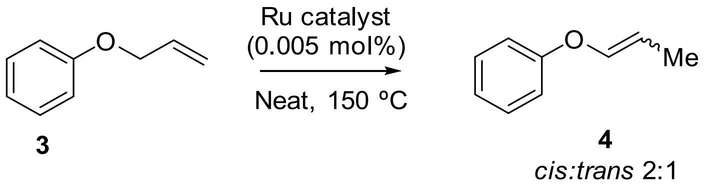

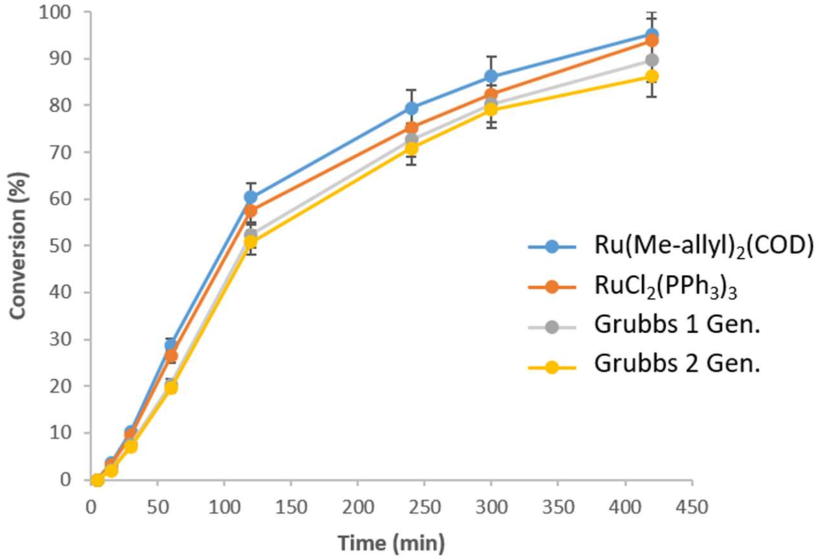

Figure S3. Kinetics for the isomerization of terminal alkene **3** to *cis/trans* internal alkenes **4** catalyzed by 50 ppm of different Ru complexes at 150 ºC. Initial rates are calculated from the linear part of the kinetic curve with maximum slope (after the induction time). Error bars account for a 5% uncertainty.

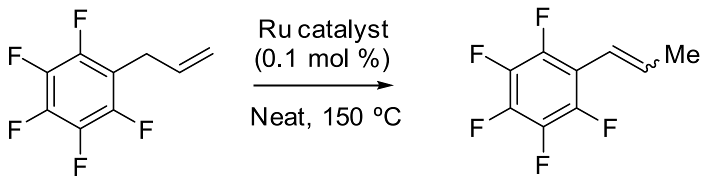

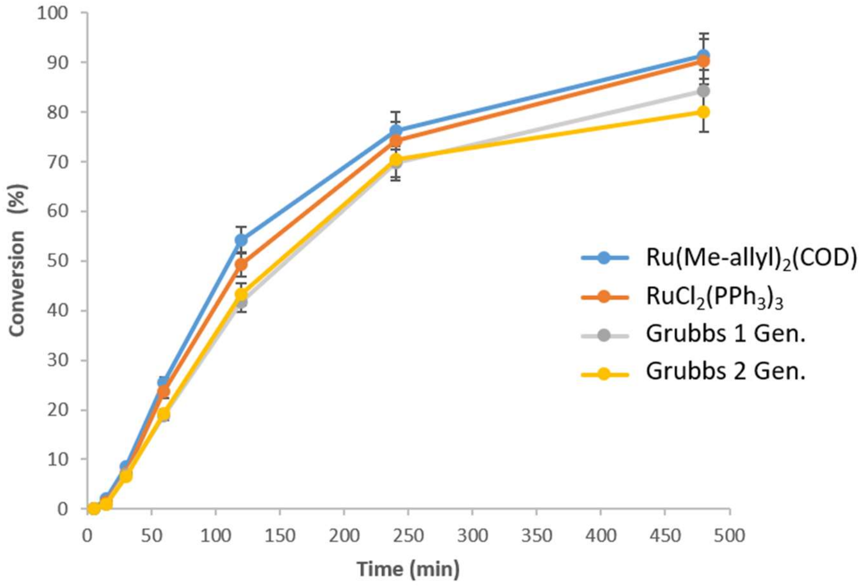

Figure S4. Kinetics for the isomerization of terminal alkene **5** to *cis/trans* internal alkenes **6** catalyzed by 1000 ppm of different Ru complexes at 150 ºC. Initial rates are calculated from the linear part of the kinetic curve with maximum slope (after the induction time). Error bars account for a 5% uncertainty.

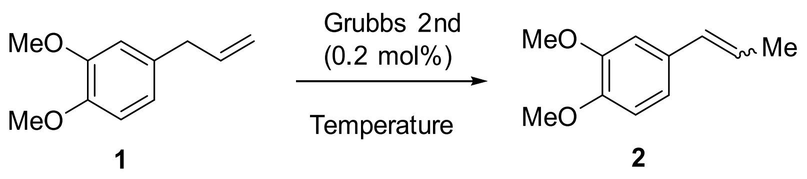

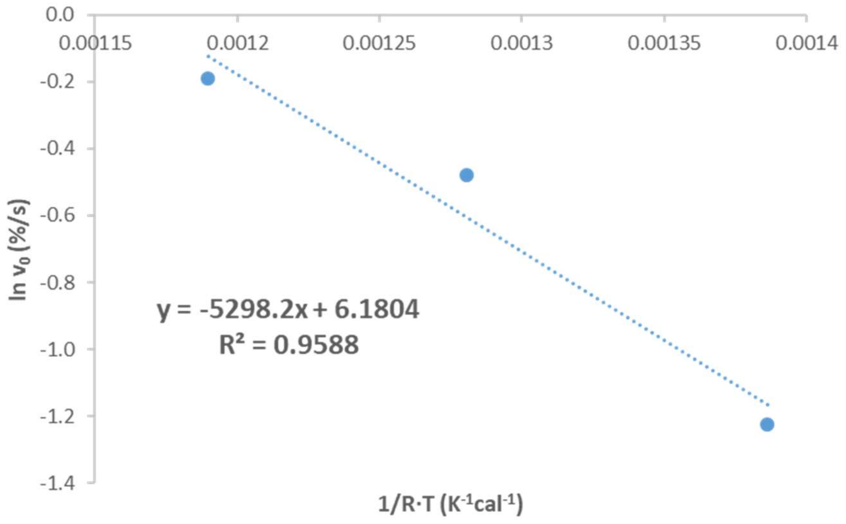

Figure S5. Eyring plot for the isomerization of methyl eugenol **1** to methyl isoeugenol **2** at different reaction temperatures. The calculated activation energy is 5.3 kcal·mol^{-1}, on the basis of both k_1 and initial rates (within a ~5% error).

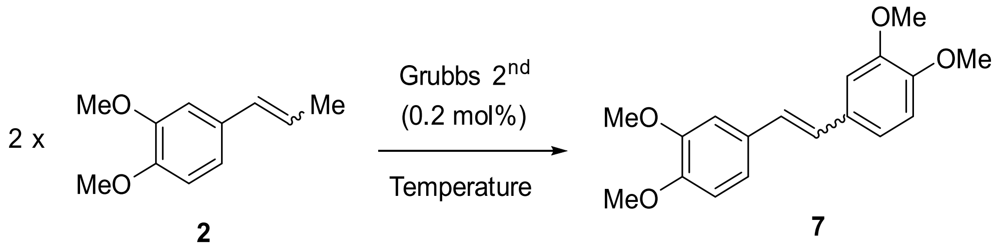

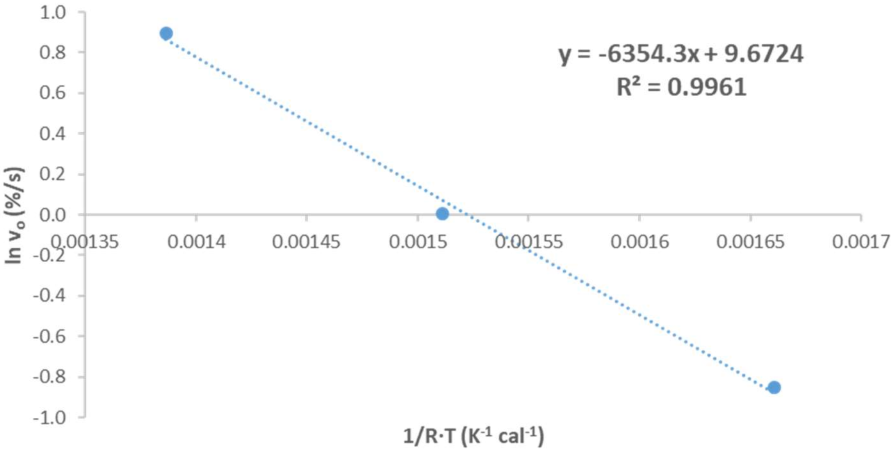

Figure S6. Eyring plot for the metathesis reaction of methyl isoeugenol **2** at different reaction temperatures. The calculated activation energy is 6.3 kcal·mol^{-1}, on the basis of both k_1 and initial rates (within a ~5% error).

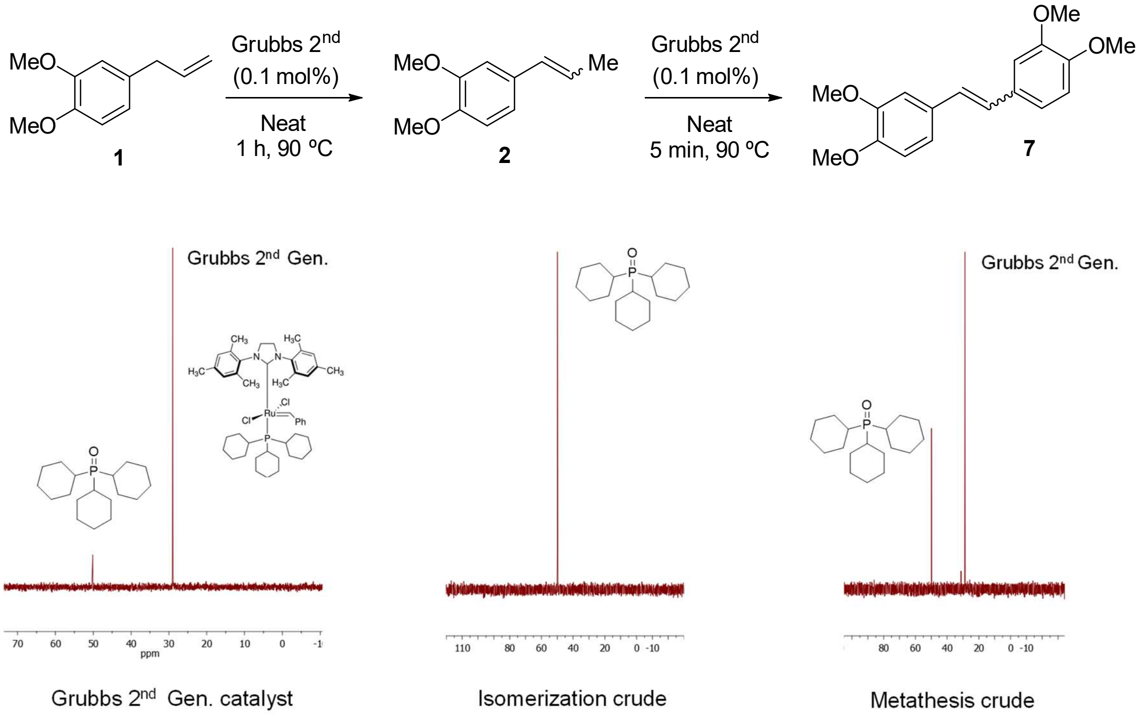

Figure S7. ^{31}P-NMR spectra of the 2nd Gen. Grubbs catalyst (structure shown) when used as a catalyst for the isomerization reaction of **1** or the metathesis reaction of **2**, under the reaction conditions indicated. The experiment was carried out in two ways, either adding fresh 2nd Gen. Grubbs catalyst after the isomerization reaction or performing an independent metathesis reaction from **2**. Both results are similar. A 10 % volume of $CDCl_3$ was added to the mixture before analysis, for shimming.

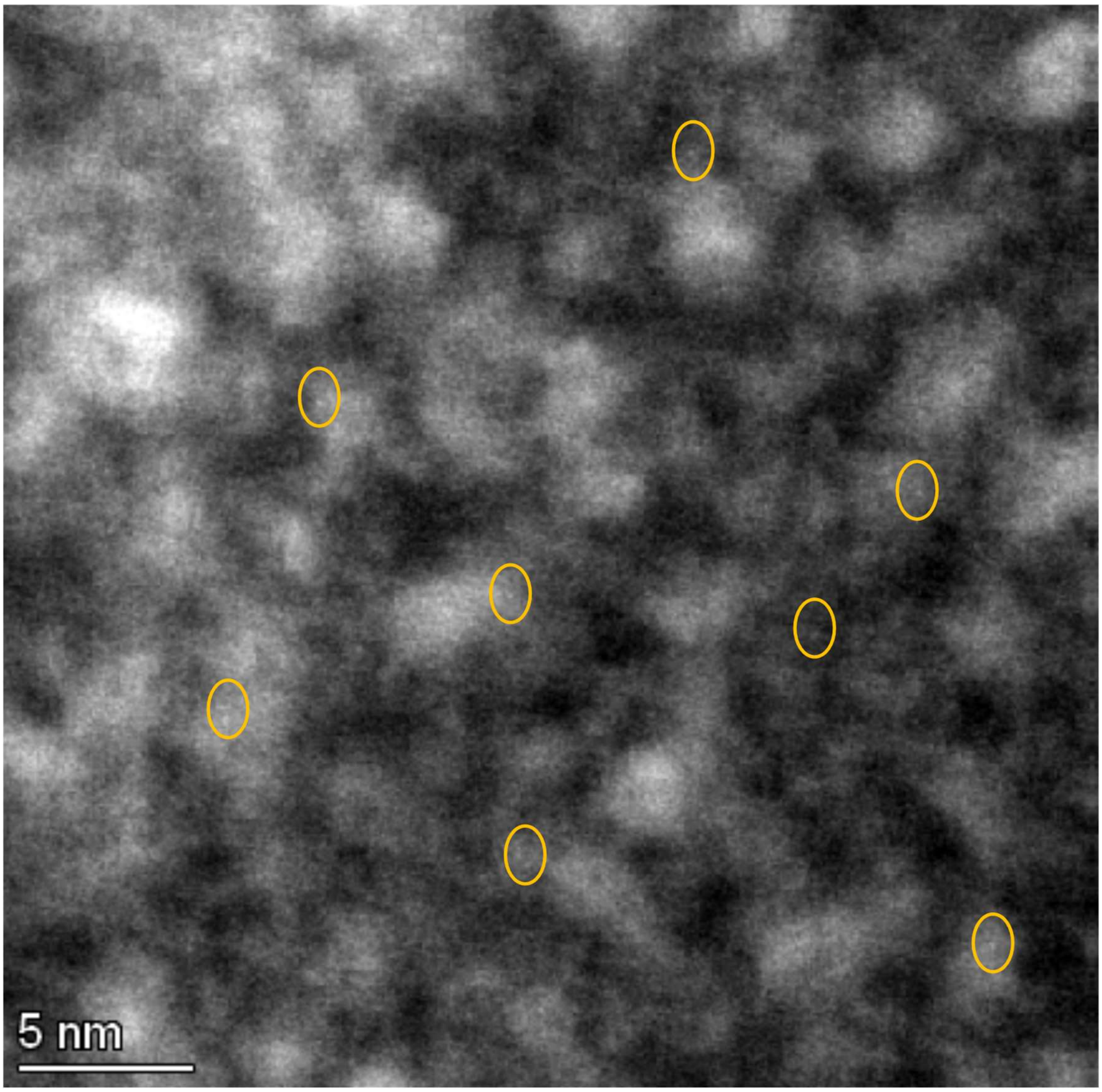

Figure S8. A representative aberration-corrected, HAADF-STEM image of the reaction solution using 0.01 mol% of $RuCl_2(PPh_3)_3$ as a pre-catalyst, during the isomerization of 1-decene **8** at 150 ºC for 2 h. Circle areas include the detected Ru atoms. Electron dispersive X-ray diffraction (EDX) experiments were also performed, and the presence of Ru was detected all over the mapping, however, EDX does not have atomic precision and is not suitable to confirm the nature of the Ru tiny spots.

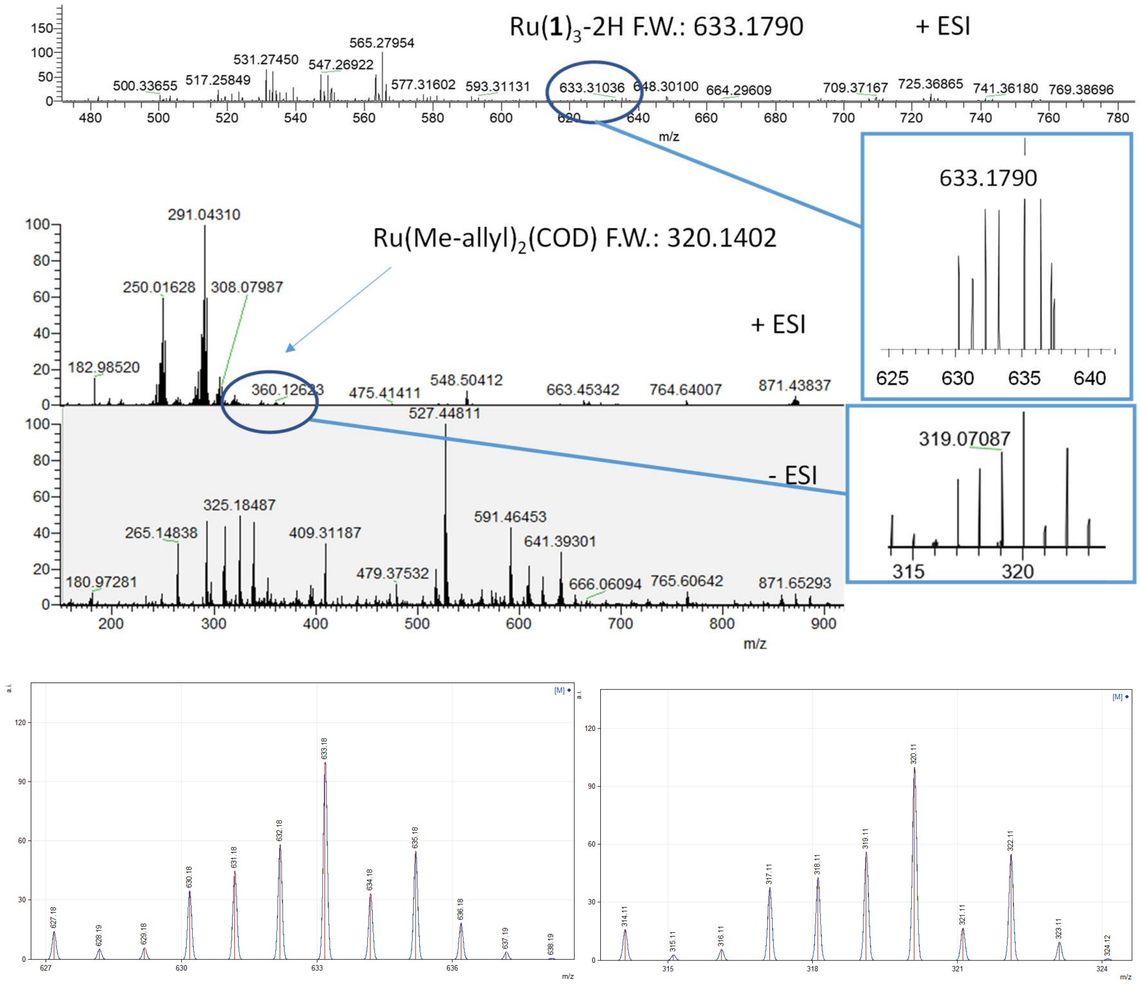

Figure S9. Top: Mass spectrometry results for the Ru-catalyzed isomerization of methyl eugenol **1** to methyl isoeugenol **2** catalyzed by 300 ppm of Ru(methyallyl)$_2$(COD), at 150 ºC. The spectra show the formation of the Ru(II) *per*alkene complex, and also show the disappearance of the neat Ru complex, in both positive and negative modes, and it can be seen that the peaks corresponding to the initial Ru complex are residual in the final mixture. Notice the correct isotopic distribution for one Ru atom in each complex found. Bottom: simulated spectra for Ru(**1**)$_3$-2H (left) and Ru(Me-allyl)$_2$COD (right), which fit well with the experimental spectra. The mass value actually fits for Ru(**1**)$_3$-2H, which could mean that the species seen is actually not Ru(0) tris alkene but rather Ru(II) bis allyl mono alkene, however, this species may be formed in the MS.

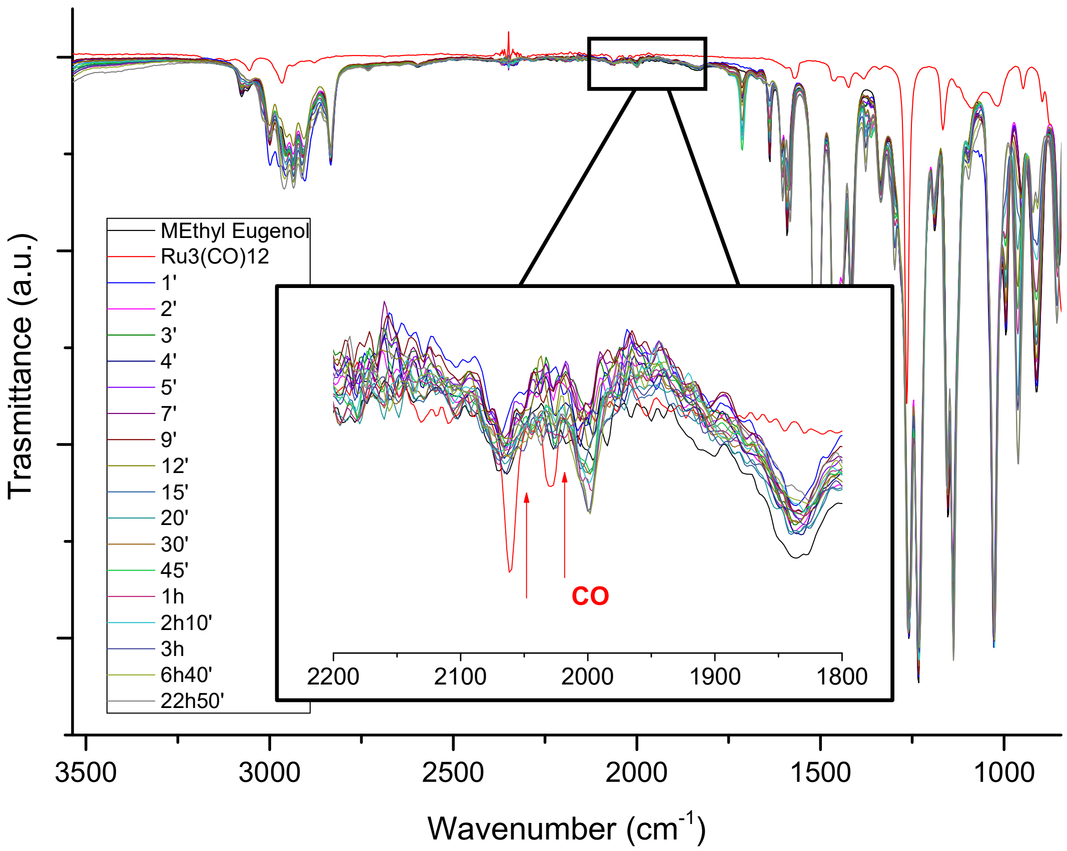

Figure S10. FT-IR spectra for the isomerization of methyl eugenol **1** (black line) to methyl isoeugenol **2** catalyzed by 300 ppm of $Ru_3(CO)_{12}$ (red line) at 150 ºC and different reaction times. The inset shows the diagnostic area, where the complete disappearance of the CO peaks from the beginning of the reaction is observed, although somewhat blurred by starting alkene traces.

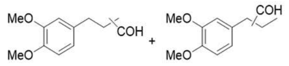

9 (different aldehydes), 0.03 %, F. W.: 194

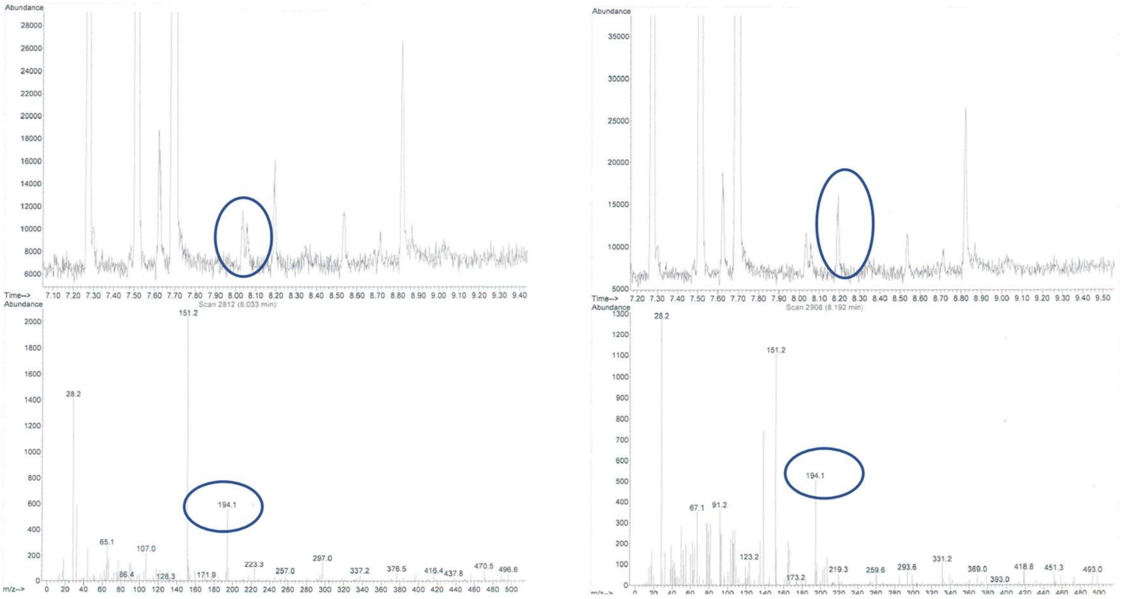

Figure S11. GC-MS spectra, where the aldehydes formed are detected.

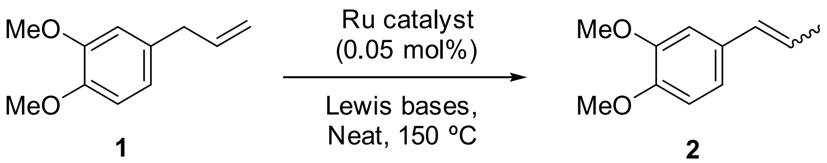

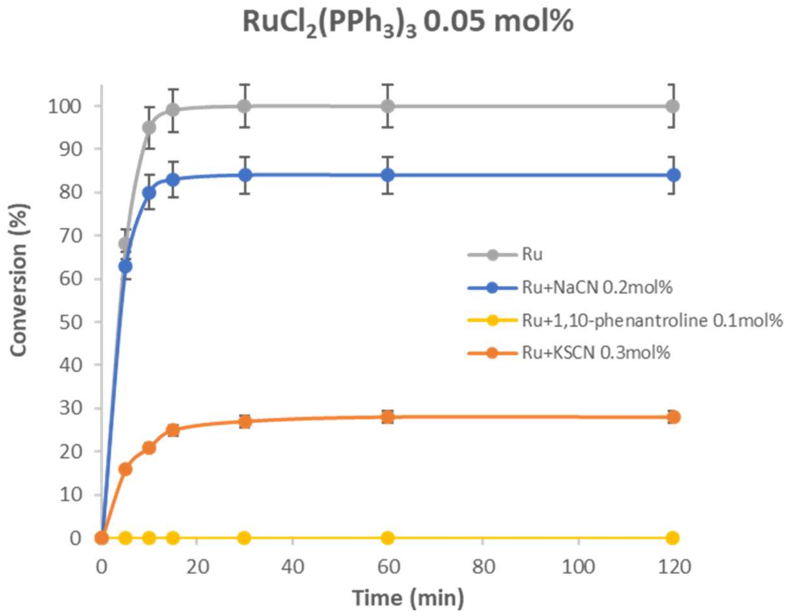

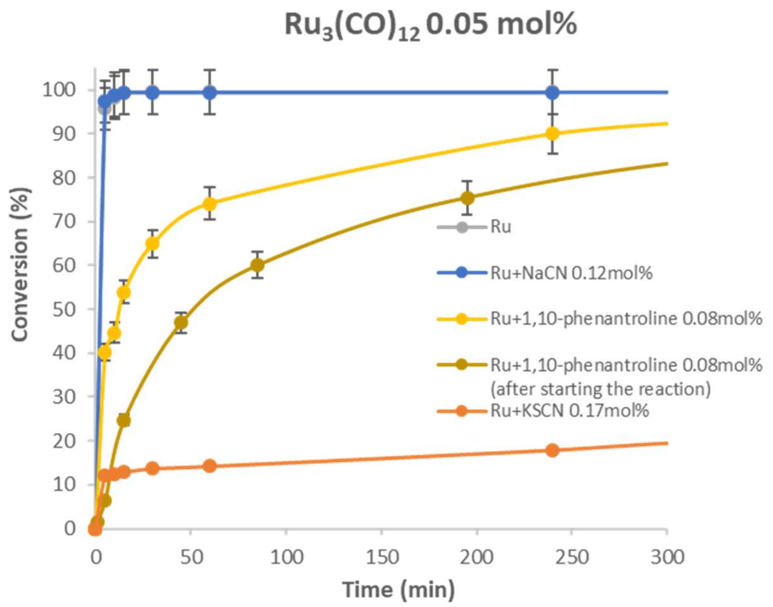

Figure S12. Poisoning experiments with NaCN, KSCN and 1,10-phenantroline added from the beginning of the reaction, for the isomerization reaction of **1** catalyzed with 0.05 mol% of $RuCl_2(PPh_3)_3$ (top) and $Ru_3(CO)_{12}$ (bottom). Initial rates are calculated from the linear part of the kinetic curve with maximum slope (after the induction time).

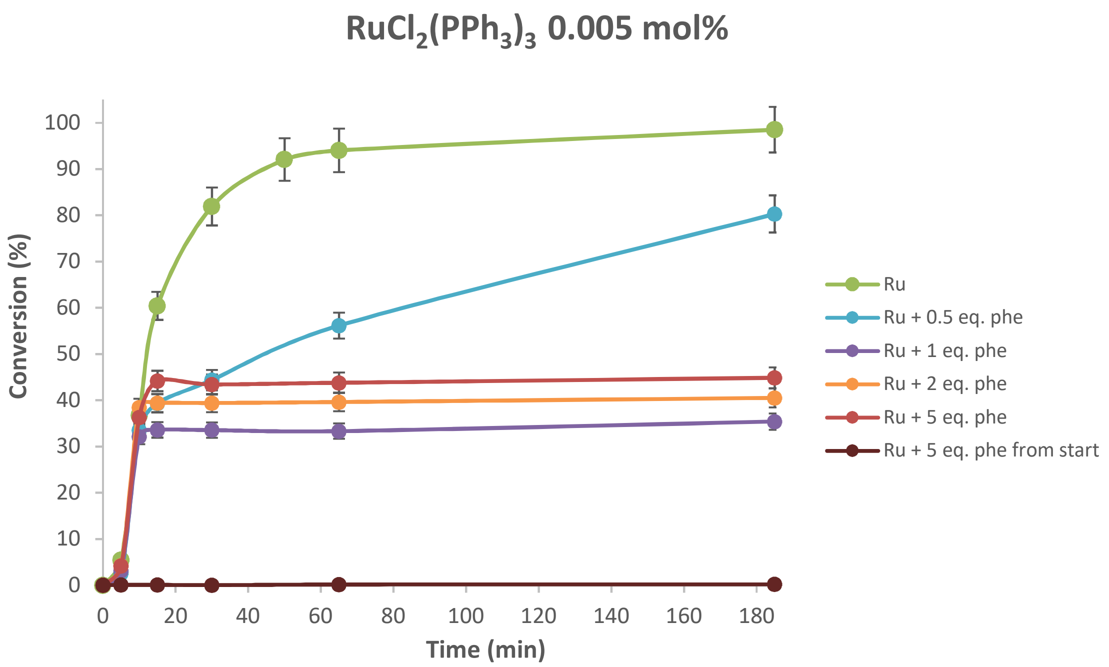

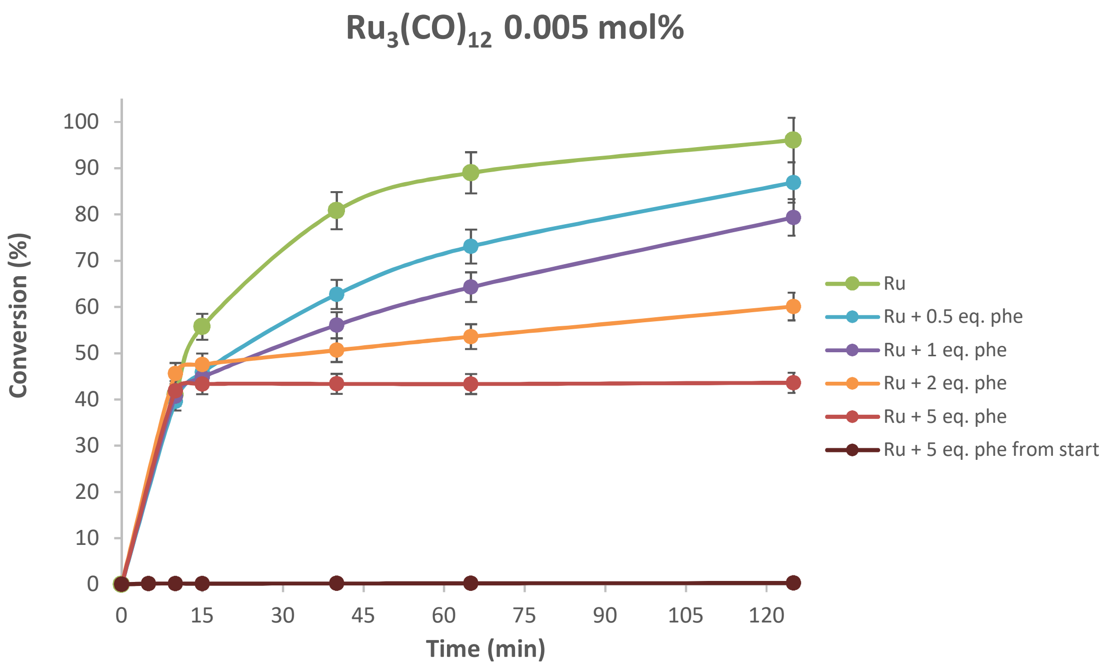

Figure S13. Poisoning experiments with 1,10-phenantroline added at 30% conversion, for the isomerization reaction of **1** catalyzed with 0.005 mol% of $RuCl_2(PPh_3)_3$ (top) and $Ru_3(CO)_{12}$ (bottom). Initial rates are calculated from the linear part of the kinetic curve with maximum slope (after the induction time).

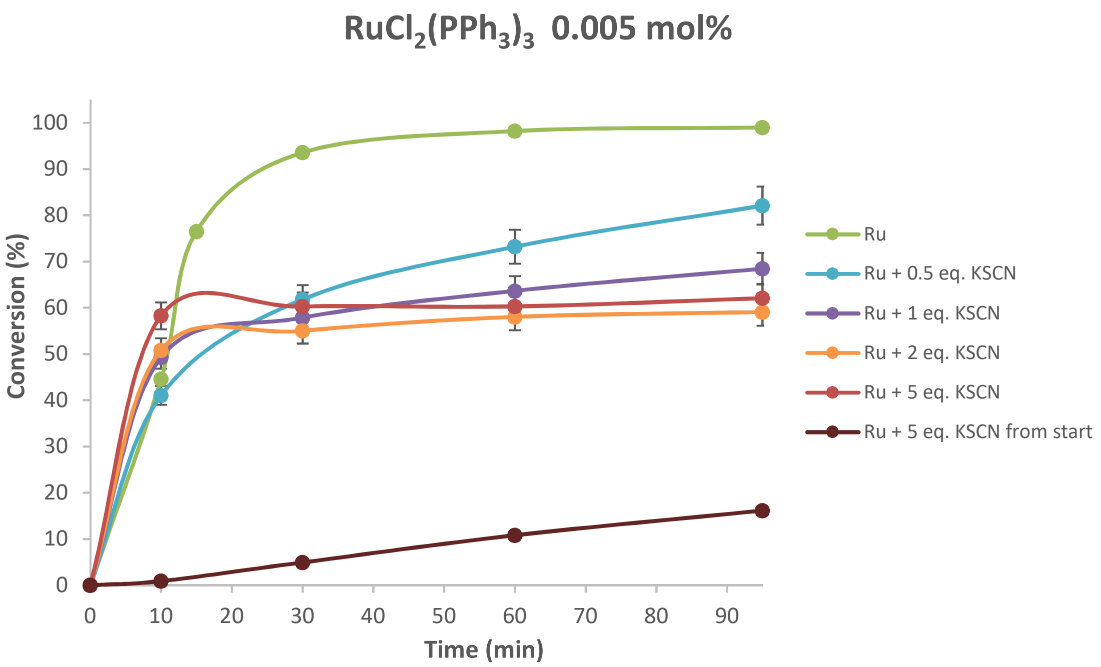

Figure S14. Poisoning experiments with KSCN added at 30% conversion, for the isomerization reaction of **1** catalyzed with 0.005 mol% of $RuCl_2(PPh_3)_3$. Initial rates are calculated from the linear part of the kinetic curve with maximum slope (after the induction time).

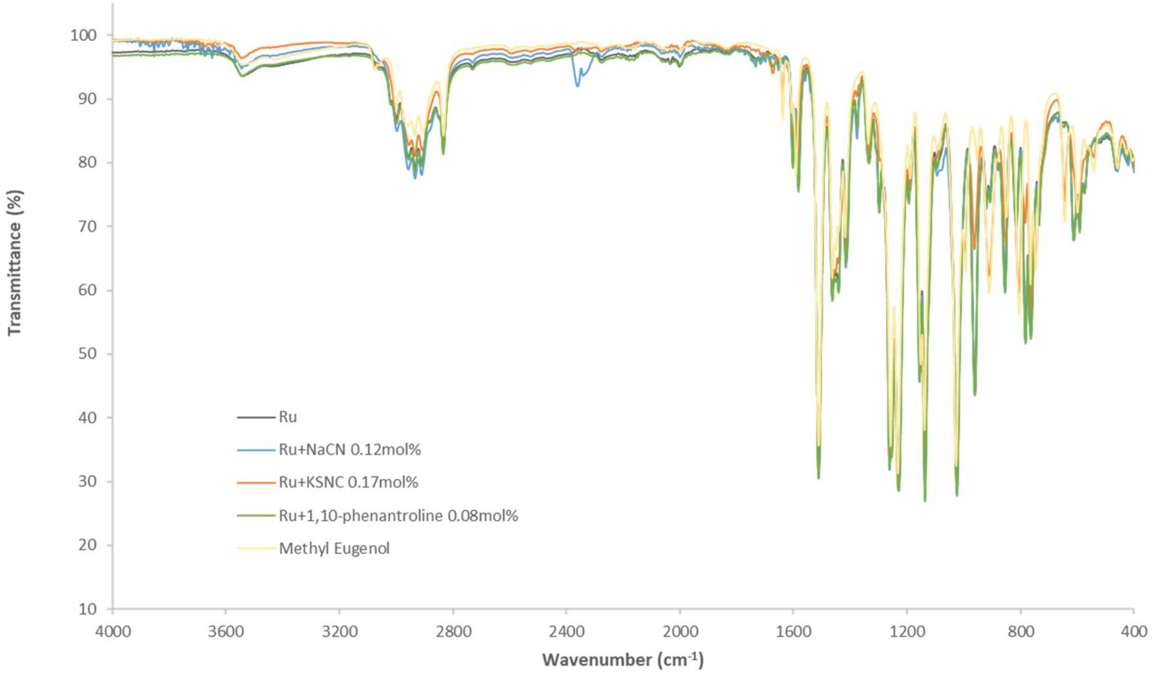

Figure S15. FT-IR of the poisoning experiments with NaCN, KSCN, 1,10-phenantroline for the isomerization reaction of **1** catalyzed with 0.05 mol% of $Ru_3(CO)_{12}$.

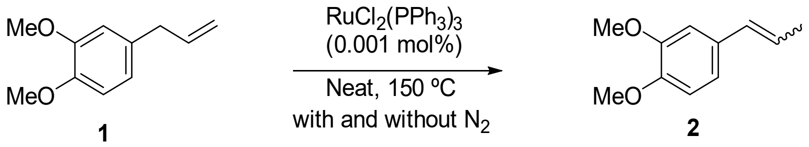

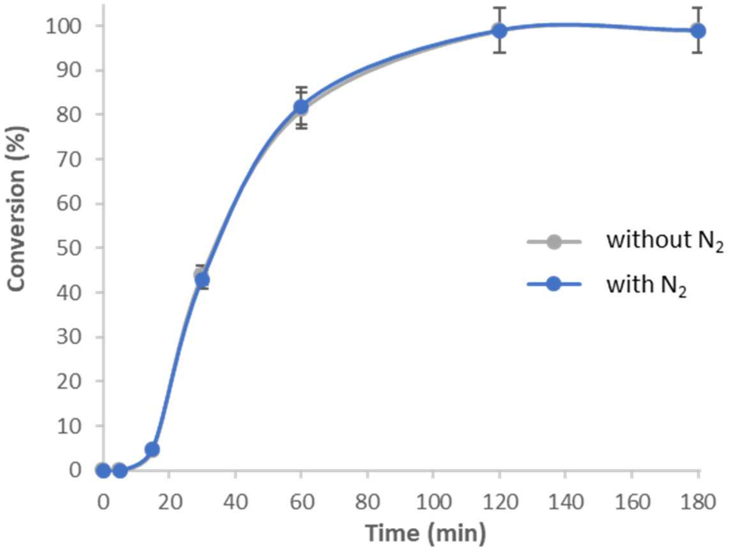

Figure S16. Kinetic results for the isomerization reaction of **1** catalyzed by 10 ppm of $RuCl_2(PPh_3)_3$ at 150 ºC under open and inert atmosphere. Error bars represent a 5% uncertainty.

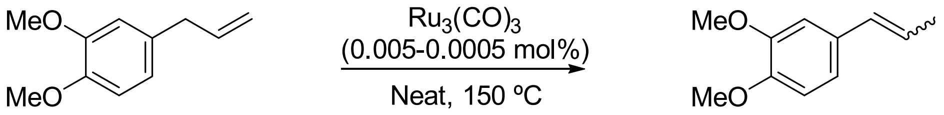

1
(5.8 mmol)

2

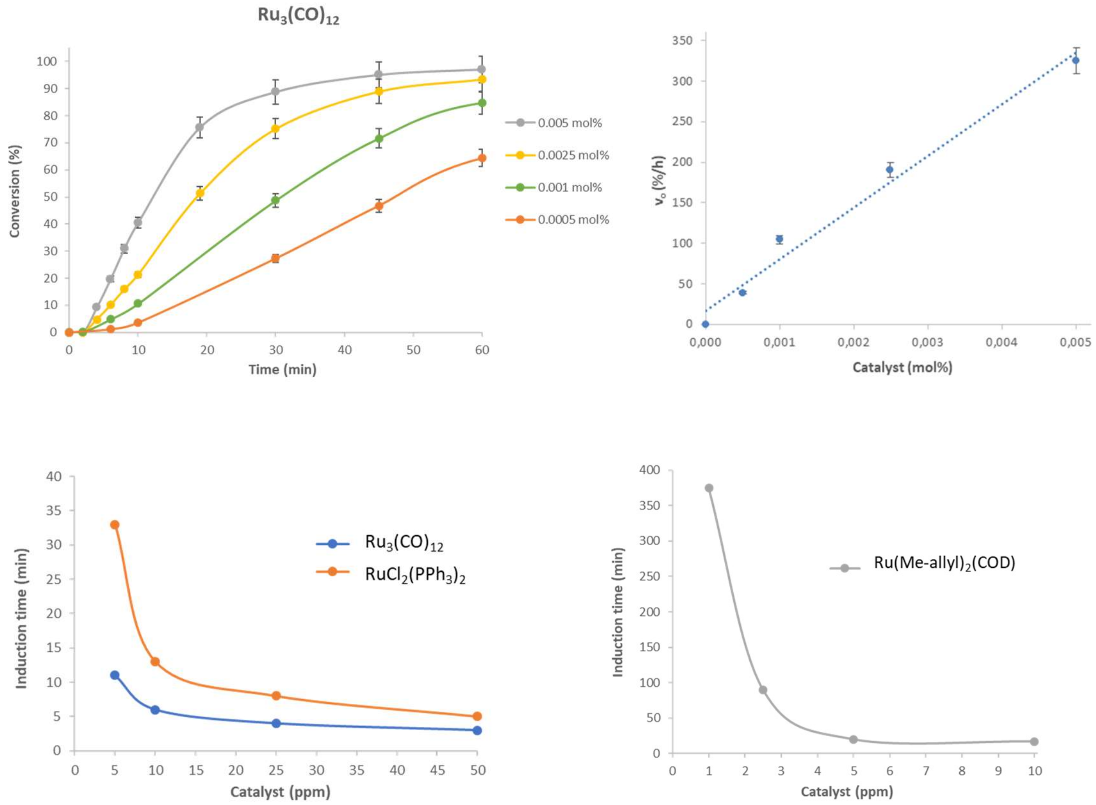

Figure S17. Kinetic plots for different amounts of $Ru_3(CO)_{12}$ catalytic precursor (top left), the correspond reaction order (top right), and correlation plots between induction time and amount of Ru precursors (bottom). Similar values are obtained when using k_1 instead of v_0. Error bars represent a 5% uncertainty.

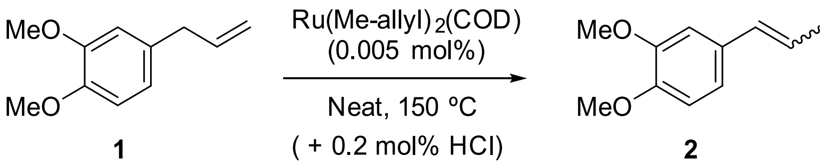

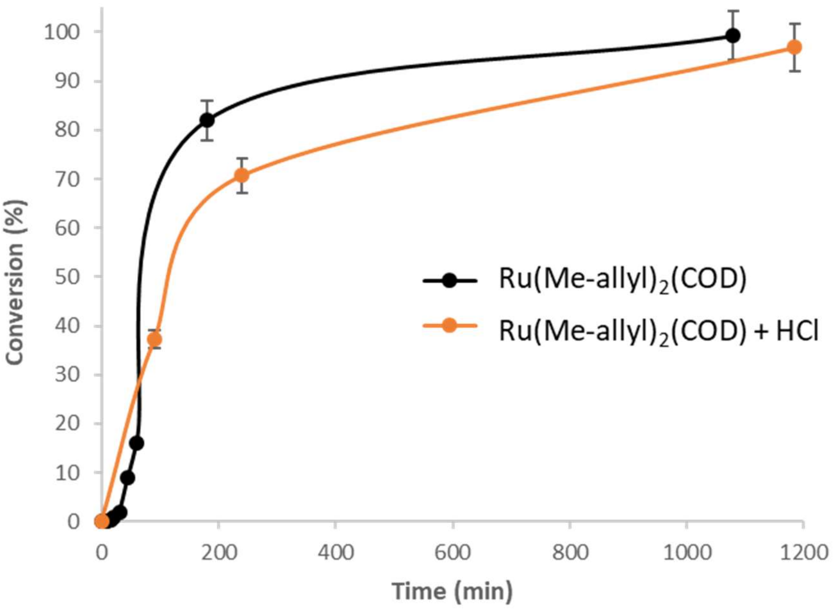

Figure S18. Influence in the isomerization reaction of the addition of HCl gas (acid) for the isomerization of methyl eugenol **1** to methyl isoeugenol **2** catalyzed by 50 ppm of Ru(methyallyl)$_2$(COD) at 150 ºC. Error bars represent a 5% uncertainty.

Compound characterization

Starting material: methyl eugenol **1**

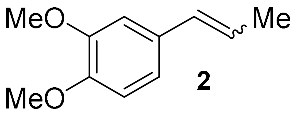

2

Composition: $C_{11}H_{14}O_2$
Molecular weight (g/mol): 178.23
Conditions: 0.001 mol% Ru(methylallyl)$_2$(COD), 150 ºC
Yield (%) (cis/trans): 97.2 (15.5/81.7)

^{1}H-NMR (401 MHz, $CDCl_3$) δ (ppm): 6.78 (s, 1H), 6.72 (d, J = 7.9 Hz, 1H), 6.65 (d, J = 7.9 Hz, 1H), 6.22 (d, J = 15.8 Hz, 1H), 6.03 – 5.94 (m, 1H), 3.75 (s, 3H), 3.72 (s, 3H), 1.75 (d, J = 6.4 Hz, 3H). ^{13}C- NMR (101 MHz, $CDCl_3$) δ (ppm): 148.98, 148.17, 131.11, 130.69, 123.57, 118.66, 111.20, 108.52, 55.78, 55.66, 18.32. IR υ (cm^{-1}): 1262.18, 1225.54 (Ar-OMe), 960.12 (=C-H).

Starting material: allyl phenyl ether **3**

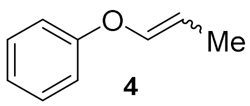

4

Composition: $C_9H_{10}O$
Molecular weight (g/mol): 134.18
Conditions: 0.005 mol% Ru(methylallyl)$_2$(COD), 150 ºC
Yield (%) (cis/trans): 92.1 (65.1/27.0)

^{1}H-NMR (401 MHz, DMSO-d_6) δ (ppm): 7.35 – 7.28 (m, 2H), 7.07 – 6.99 (m, 3H), 6.56 (m, 1H), 4.91 – 4.82 (m, 1H), 1.65 (d, J = 6.8 Hz, 3H). ^{13}C- NMR (101 MHz, DMSO-d_6) δ (ppm): 157.36, 141.40, 130.18, 122.85, 116.30, 106.84, 9.72. IR υ (cm^{-1}): 1222.65 (Ar-OR), 929.52 (=C-H).

Starting material: allylpentafluorobenzene **5**

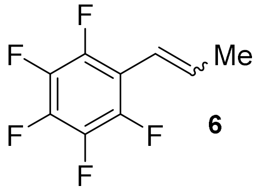

6

Composition: $C_9H_5F_5$
Molecular weight (g/mol): 208.13
Conditions: 0.1 mol% Ru(methylallyl)$_2$(COD), 150 ºC
Yield (%) (cis/trans): 91.6 (2.8/88.8)

^{1}H-NMR (401 MHz, DMSO-d_6) δ (ppm): 6.51 (dq, J = 16.1, 6.7 Hz, 1H), 6.23 (d, J = 16.2 Hz, 1H), 1.90 (dd, J = 6.7, 0.8 Hz, 3H). ^{13}C- NMR (101 MHz, DMSO-d_6) δ (ppm): 146.11, 142.80, 141.19 – 140.65, 139.27, 137.58, 115.29, 112.50. ^{19}F-NMR (377 MHz, $CDCl_3$) δ (ppm): -144.10 (dd, J = 21.5, 7.7 Hz, 2F), -158.11 (t, J = 20.8 Hz, 1F), -163.56 (dt, J = 21.2, 7.7 Hz, 2F). IR υ (cm^{-1}): 911.33 (=C-H).

Starting material: methyl eugenol **1**

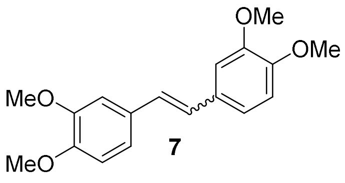

Composition: $C_{18}H_{20}O_4$
Molecular weight (g/mol): 300.14
Conditions: 0.1 mol% Grubbs 2nd Gen., 90 ºC
Yield (%): 94.4

^{1}H-NMR (401 MHz, DMSO-d_6) δ (ppm): 7.00 (d, J = 1.9 Hz, 2H), 6.97 (dd, J = 8.2, 1.9 Hz, 2H), 6.86 (s, 2H), 6.79 (d, J = 8.2 Hz, 2H), 3.89 (s, 6H), 3.84 (s, 6H). ^{13}C- NMR (101 MHz, DMSO-d_6) δ (ppm): 149.14, 148.71, 130.69, 126.65, 119.57, 111.27, 108.60, 55.96, 55.87. IR υ (cm^{-1}): 1262.18, 1262.17 ,1239.04, 1230,36 (Ar-OMe).

Starting material: 1-decene **8**

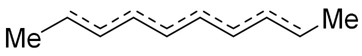

9

Composition: $C_{10}H_{20}$
Molecular weight (g/mol): 140.16
Conditions: 0.01 mol% $RuCl_2(PPh_3)_3$, 150 ºC
Yield (%): 94.0

^{1}H-NMR (401 MHz, DMSO-d_6) δ (ppm): (2-Alkene) 5.55 – 5.29 (m, 2H), 2.09 – 1.89 (m, 2H), 1.62 (m, 3H), 1.43 – 1.19 (m, 10H), 0.99 – 0.81 (m, 3H). ^{13}C- NMR (101 MHz, DMSO-d_6) δ (ppm): (2-Alkene) 131.77, 124.48, 32.57, 31.81, 29.77, 29.33 ,28.69, 22.64, 17.86, 14.06. IR υ (cm^{-1}): 964.23 (=C-H).

NMR copies.

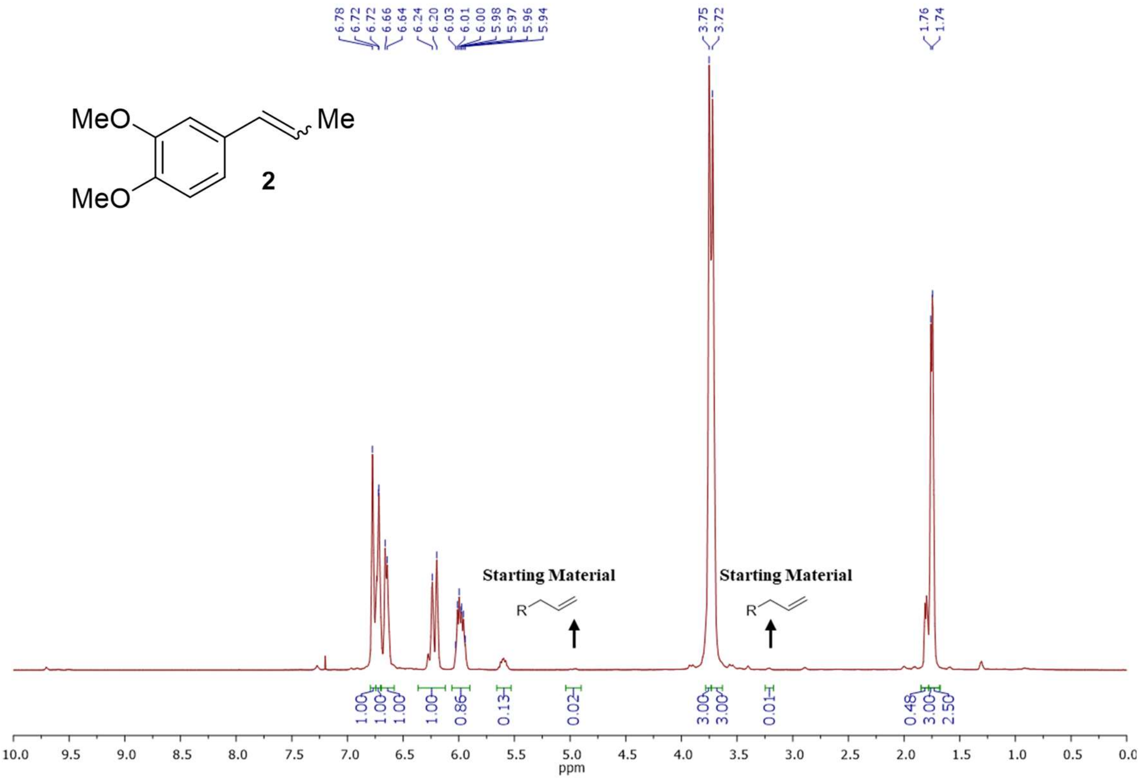

MeO
MeO
Me
2
6.78
6.72
6.72
6.66
6.64
6.24
6.20
6.03
6.01
6.00
5.98
5.97
5.96
5.94
3.75
3.72
1.76
1.74
Starting Material
Starting Material
1.00
1.00
1.00
1.00
0.86
0.13
0.02
3.00
3.00
0.01
0.48
3.00
2.50
ppm

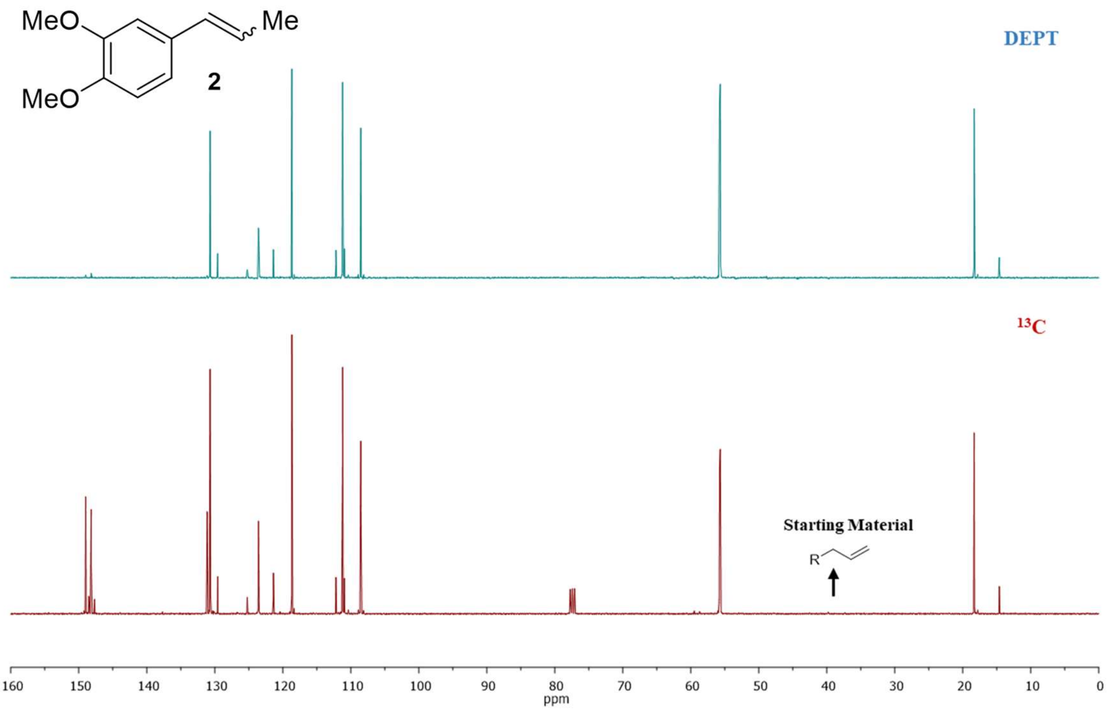

MeO
MeO
Me
2
DEPT
¹³C
Starting Material
ppm

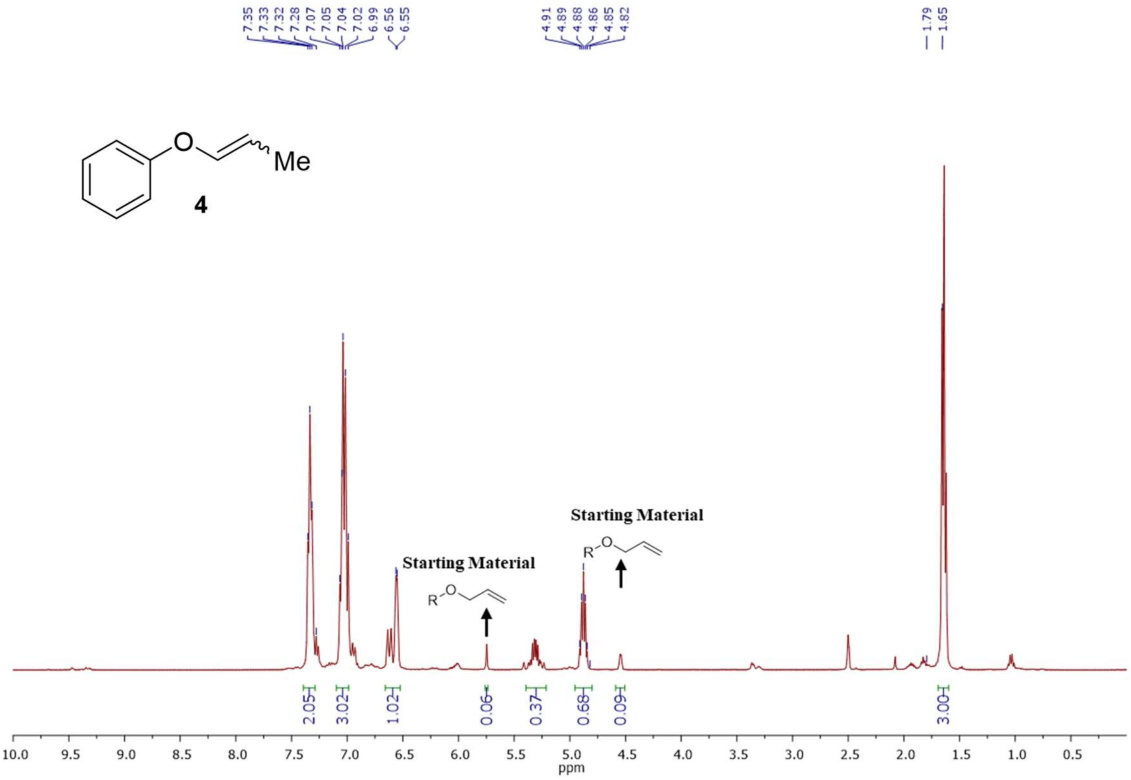
7.35 7.33 7.32 7.28 7.07 7.05 7.04 7.02 6.99 6.56 6.55
4.91 4.89 4.88 4.86 4.85 4.82
1.79 1.65
O
Me
4
Starting Material
R
O
Starting Material
R
O
2.05 3.02 1.02 0.06 0.37 0.68 0.09 3.00
10.0 9.5 9.0 8.5 8.0 7.5 7.0 6.5 6.0 5.5 5.0 4.5 4.0 3.5 3.0 2.5 2.0 1.5 1.0 0.5
ppm

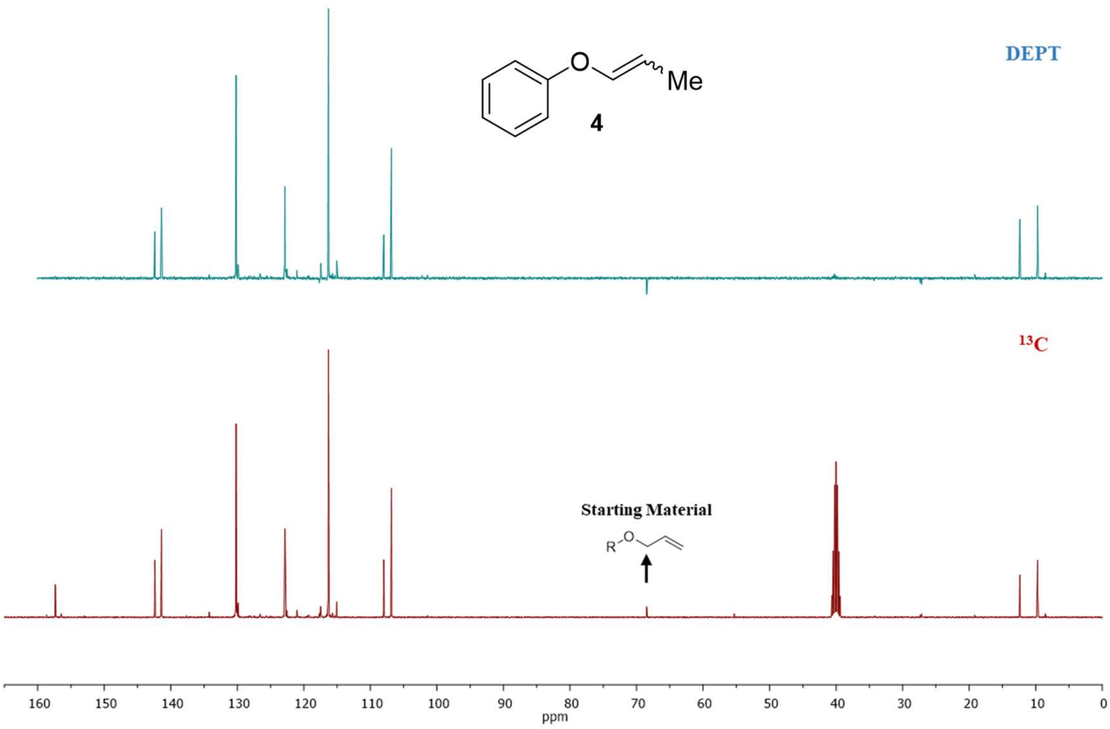
O
Me
4
DEPT
^{13}C
Starting Material
R
O
160 150 140 130 120 110 100 90 80 70 60 50 40 30 20 10 0
ppm

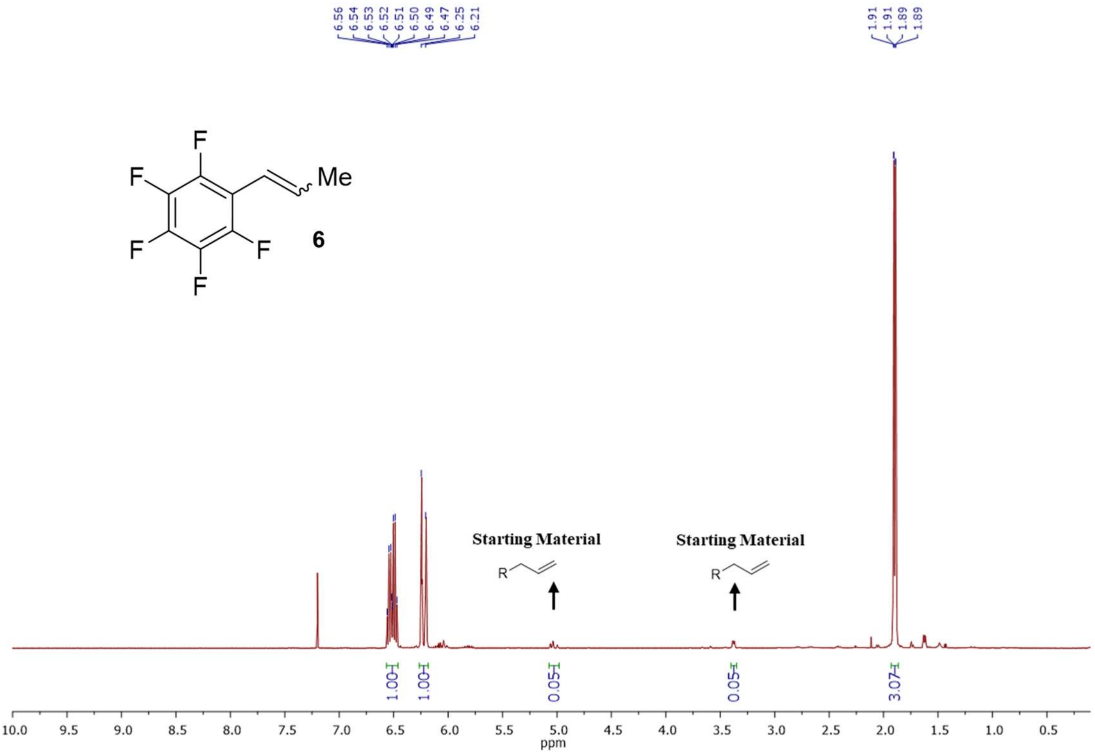
6.56
6.54
6.53
6.52
6.51
6.50
6.49
6.47
6.25
6.21
1.91
1.91
1.89
1.89
F
F
F
F
F
Me
6
Starting Material
R
Starting Material
R
1.00
1.00
0.05
0.05
3.07
10.0
9.5
9.0
8.5
8.0
7.5
7.0
6.5
6.0
5.5
5.0
4.5
4.0
3.5
3.0
2.5
2.0
1.5
1.0
0.5
ppm

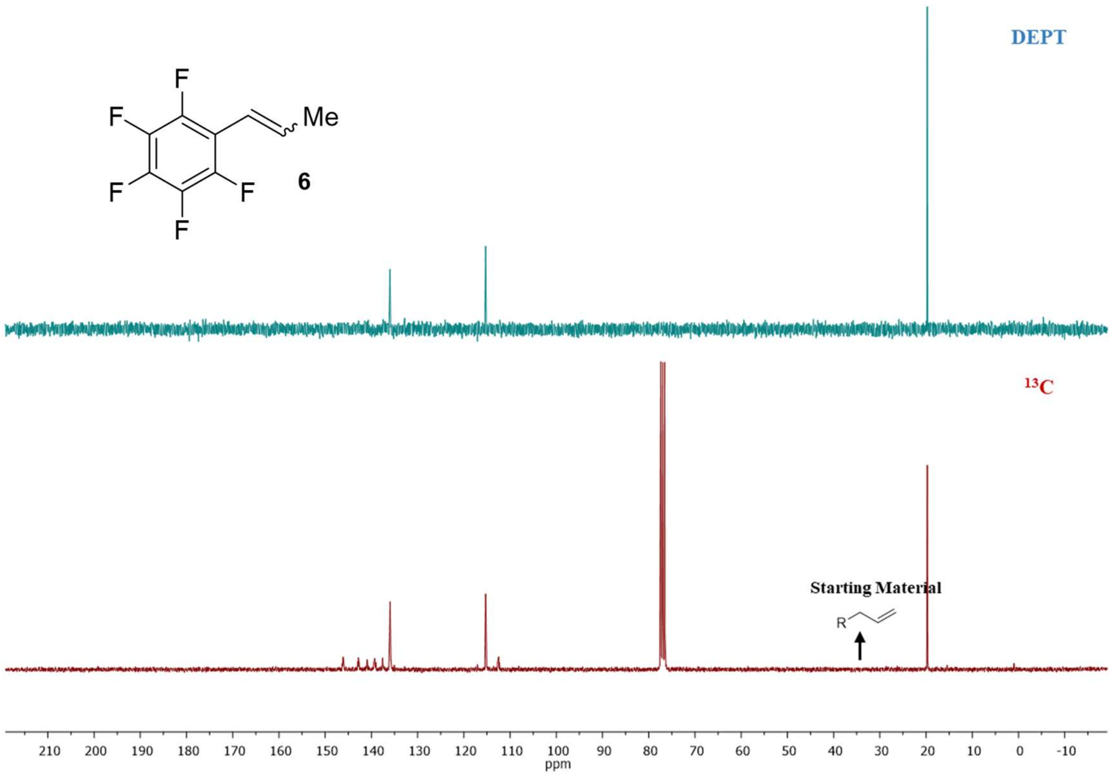
DEPT
F
F
F
F
F
Me
6
13C
Starting Material
R
210
200
190
180
170
160
150
140
130
120
110
100
90
80
70
60
50
40
30
20
10
0
-10
ppm

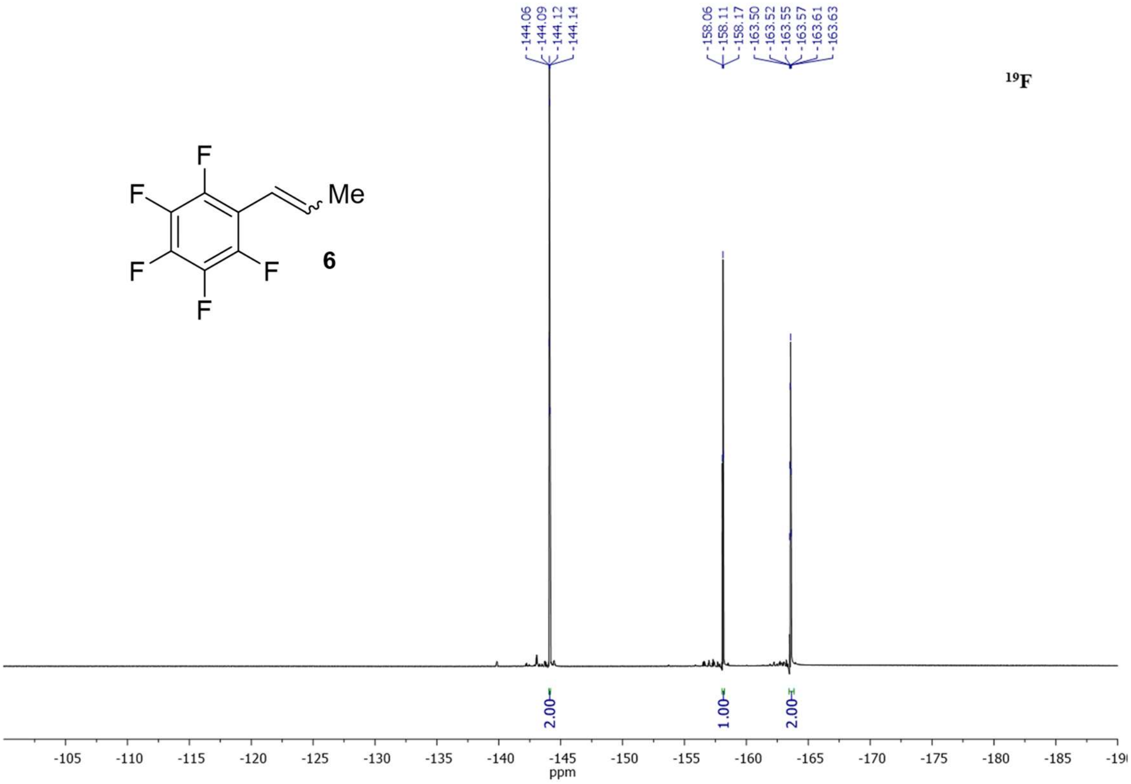
-144.06
-144.09
-144.12
-144.14
-158.06
-158.11
-158.17
-163.50
-163.52
-163.55
-163.57
-163.61
-163.63
¹⁹F
F
F
F
F
F
Me
6
2.00
1.00
2.00
-105 -110 -115 -120 -125 -130 -135 -140 -145 -150 -155 -160 -165 -170 -175 -180 -185
ppm

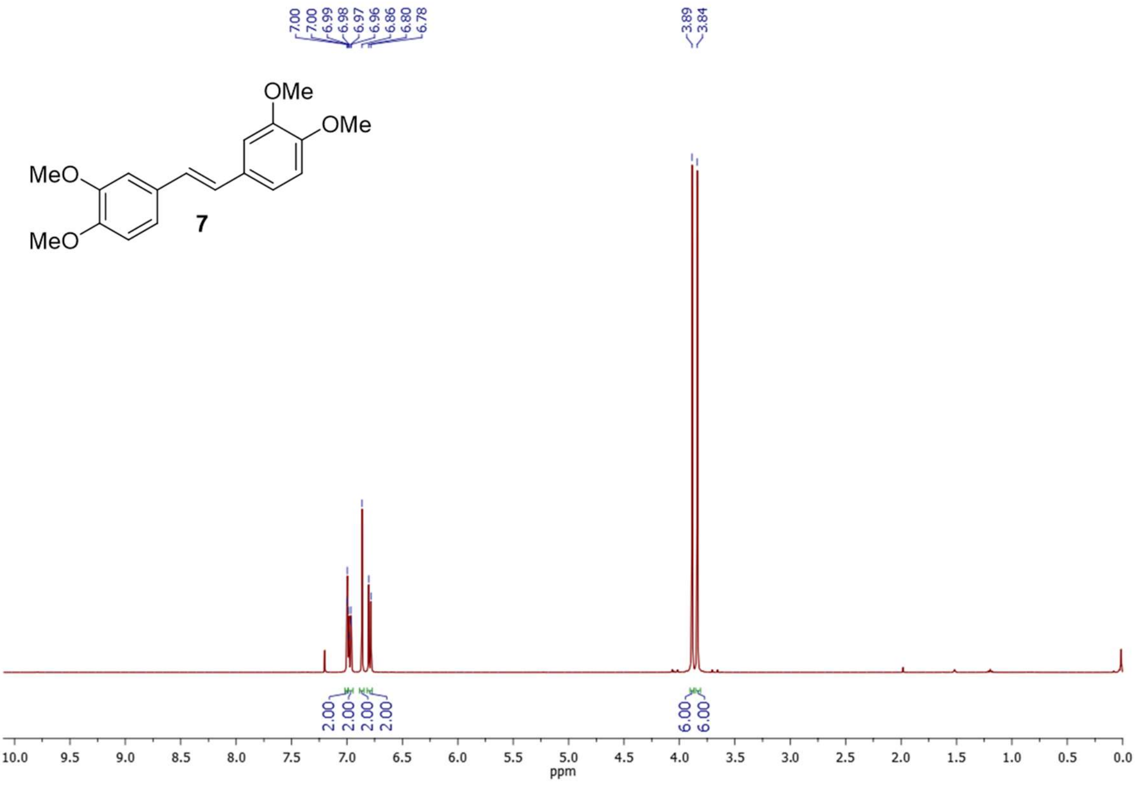
7.00
7.00
6.99
6.98
6.97
6.96
6.86
6.80
6.78
3.89
3.84
OMe
OMe
MeO
MeO
7
2.00
2.00
2.00
2.00
6.00
6.00
10.0 9.5 9.0 8.5 8.0 7.5 7.0 6.5 6.0 5.5 5.0 4.5 4.0 3.5 3.0 2.5 2.0 1.5 1.0 0.5 0.0
ppm

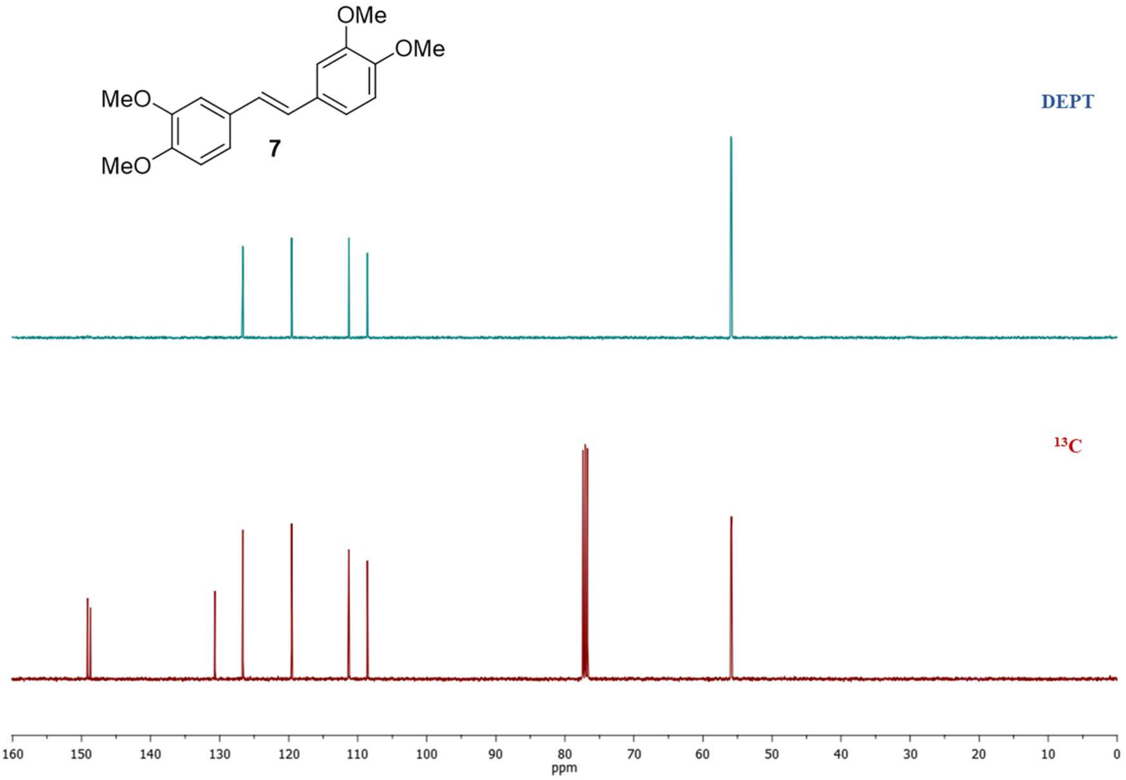
OMe
OMe
MeO
MeO
7
DEPT
13C
160 150 140 130 120 110 100 90 80 70 60 50 40 30 20 10 0
ppm

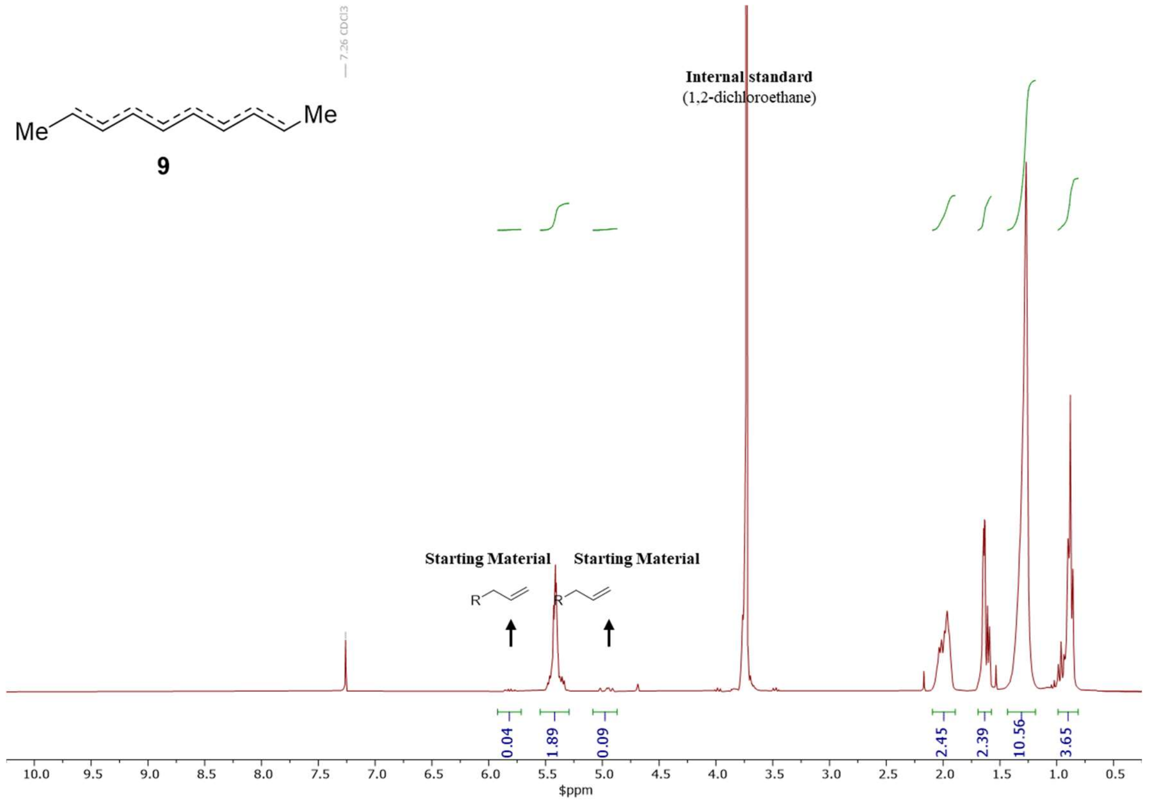
7.26 CDCl3
Me
Me
9
Internal standard
(1,2-dichloroethane)
Starting Material
R
Starting Material
R
0.04
1.89
0.09
2.45
2.39
10.56
3.65
10.0 9.5 9.0 8.5 8.0 7.5 7.0 6.5 6.0 5.5 5.0 4.5 4.0 3.5 3.0 2.5 2.0 1.5 1.0 0.5
$ppm

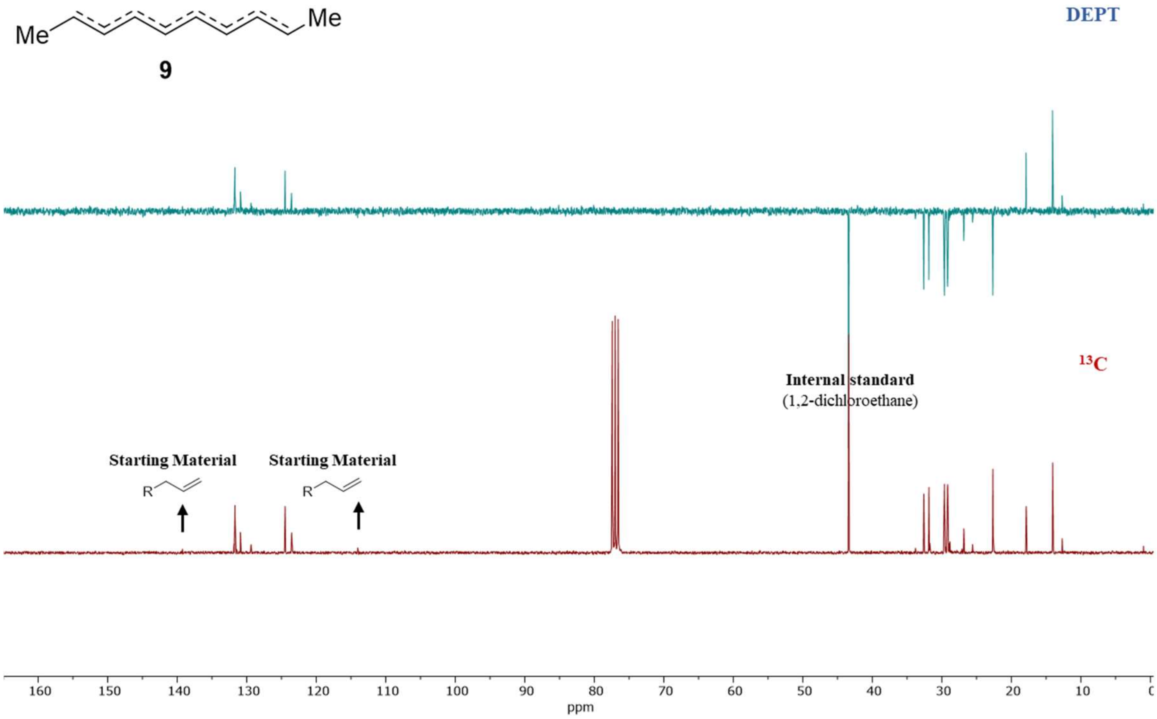
Me
Me
9
DEPT
13C
Internal standard
(1,2-dichloroethane)
Starting Material
Starting Material
R
R
160
150
140
130
120
110
100
90
80
70
60
50
40
30
20
10
ppm
